# A gain-of-function screen to identify genes that reduce lifespan in the adult of *Drosophila melanogaster*

**DOI:** 10.1186/1471-2156-15-46

**Published:** 2014-04-16

**Authors:** Minoru Nakayama, Tomoki Ishibashi, Hiroyuki O Ishikawa, Hiroyasu Sato, Takao Usui, Takayuki Okuda, Hiroyuki Yashiro, Hironori Ishikawa, Yoshie Taikou, Asako Minami, Kengo Kato, Masataka Taki, Toshiro Aigaki, Wataru Gunji, Masaya Ohtsu, Yasufumi Murakami, Sei-ichi Tanuma, Alice Tsuboi, Mai Adachi, Junpei Kuroda, Takeshi Sasamura, Tomoko Yamakawa, Kenji Matsuno

**Affiliations:** 1 Genome and Drug Research Center, Tokyo University of Science, Noda, Chiba 278-8510, Japan; 2 Department of Biological Sciences, Osaka University, 1-1 Machikaneyama, Toyonaka, Osaka 560-0043, Japan; 3 Department of Biological Science and Technology, Tokyo University of Science, Noda, Chiba 278-8510, Japan; 4 Department of Biological Sciences, Tokyo Metropolitan University, Hachioji, Tokyo 192-0397, Japan; 5 Department of Molecular Biosciences, Faculty of Life Sciences, Kyoto Sangyo University, Kita-ku, Kyoto 603-8555, Japan; 6 Graduate School of Science, Chiba University, Chiba, Chiba 263-8522, Japan

**Keywords:** Genetic screen, *Drosophila*, Apoptosis, Reduced lifespan, Gene misexpression

## Abstract

**Background:**

Several lines of evidence associate misregulated genetic expression with risk factors for diabetes, Alzheimer’s, and other diseases that sporadically develop in healthy adults with no background of hereditary disorders. Thus, we are interested in genes that may be expressed normally through parts of an individual’s life, but can cause physiological defects and disease when misexpressed in adulthood.

**Results:**

We attempted to identify these genes in a model organism by arbitrarily misexpressing specific genes in adult *Drosophila melanogaster*, using 14,133 Gene Search lines. We identified 39 “reduced-lifespan genes” that, when misexpressed in adulthood, shortened the flies’ lifespan to less than 30% of that of control flies. About half of these genes have human orthologs that are known to be involved in human diseases. For about one-fourth of the reduced-lifespan genes, suppressing apoptosis restored the lifespan shortened by their misexpression. We determined the organs responsible for reduced lifespan when these genes were misexpressed specifically in adulthood, and found that while some genes induced reduced lifespan only when misexpressed in specific adult organs, others could induce reduced lifespan when misexpressed in various organs. This finding suggests that tissue-specific dysfunction may be involved in reduced lifespan related to gene misexpression. Gene ontology analysis showed that reduced-lifespan genes are biased toward genes related to development.

**Conclusions:**

We identified 39 genes that, when misexpressed in adulthood, shortened the lifespan of adult flies. Suppressing apoptosis rescued this shortened lifespan for only a subset of the reduced-lifespan genes. The adult tissues in which gene misexpression caused early death differed among the reduced-lifespan genes. These results suggest that the cause of reduced lifespan upon misexpression differed among the genes.

## Background

Major risk factors for late-onset sporadic diseases such as diabetes, inflammation, neurodegeneration, and cancer are believed to include aging, unhealthy lifestyles, and various stresses [1-3]. However, the molecular mechanisms that increase morbidity in these diseases are not completely understood. Shortened telomeres is a factor in cellular aging and genomic instabilities, and could increase susceptibility to cancer [3]. It has also been proposed that gene expression levels are altered by aging or various stresses, and these changes may account for triggers or risk factors of late-onset diseases. For example, Calpain, a Ca^2+^-activated cysteine protease that accumulates in the brain of Parkinson’s disease patients [4], is induced by various stimuli including oxidative stress [5]. Upregulated Calpain activity has also been linked to neurodegenerative diseases such as Alzheimer’s disease and Parkinson’s disease [4,6]. Alterations in gene expression levels in response to age or stress have been observed in model animals; in the mouse brain, for example, several genes involved in immune or inflammatory responses are upregulated with aging [7]. In *Drosophila*, the expression levels of many genes change with aging and in respond to oxidative stress [8]. However, the potential impact of these changes remains to be clarified, even in these model organisms.

The *Drosophila melanogaster* genome project revealed that 61% of the known human disease genes are conserved between humans and *Drosophila*[9,10]. The short life cycle and the powerful tools for genetic and molecular analysis available in *Drosophila* make this species an advantageous model for studying the functions of genes associated with various human diseases [11,12]. In particular, mutants of *Drosophila* homologs of human disease genes allow us to study the developmental, cellular, and molecular functions of these genes [13,14]. For example, *Drosophila* models of Parkinson’s disease have provided important insights into the relationships among genes that mediate this disease in humans [15]. However, most studies of mutations in *Drosophila* homologs of human disease genes have focused on developmental defects.

Since genes that strongly affect physiology and homeostasis when their expression levels are altered could account for the increasing morbidity rate of late-onset diseases in humans, and expression levels can be altered by aging and other stresses, it is worthwhile to examine the impact of changes in gene expression at the adult stage of model organisms such as *Drosophila*. The consequences of genetic perturbations that occur after reaching adulthood may help us understand how aberrant gene expression relates to the appearance of late-onset defects and loss of longevity. Therefore, using the *Drosophila* system, we attempted to identify genes that reduce lifespan when misexpressed only in adulthood.

The *Drosophila* gene search (GS) line allows us to spatially and temporally control the expression of specific genes in the genome [16]. Gain-of-function screens using the GS system have revealed new components in biological processes such as the determination of tissue identity, neural cell death, neural development, and longevity [17-21]. Here, using the GS system, we identified and characterized 39 genes that severely reduced longevity when misexpressed in adulthood.

## Results and discussion

### Primary screen

This study was designed to identify genes that severely reduced the *Drosophila* lifespan when misexpressed in adulthood. To accomplish this, we arbitrarily misexpressed genes in adult flies from various GS lines [16]. Each GS line carries a GS vector, an engineered *Drosophila* transposon that carries a promoter (GS promoter) controlled by the UAS. The GS promoter contains binding sites for a yeast transcription factor, GAL4 [22]. The GS promoter is activated in the presence of GAL4, and its transcription activity is negligible without GAL4 [22]. Because the GS vector does not have a transcription terminator sequence, mRNA precursor synthesis continues through the endogenous gene next to the GS vector insertion site. Thus, in each GS line, one endogenous gene—which can be predicted based on the GS vector insertion site—is misexpressed in a GAL4-dependent manner [16]. In this study, we screened 14,133 GS lines with the potential to misexpress 4,605 genes (DGSP, http://gsdb.biol.se.tmu.ac.jp/~dclust/).

**Figure 1:**
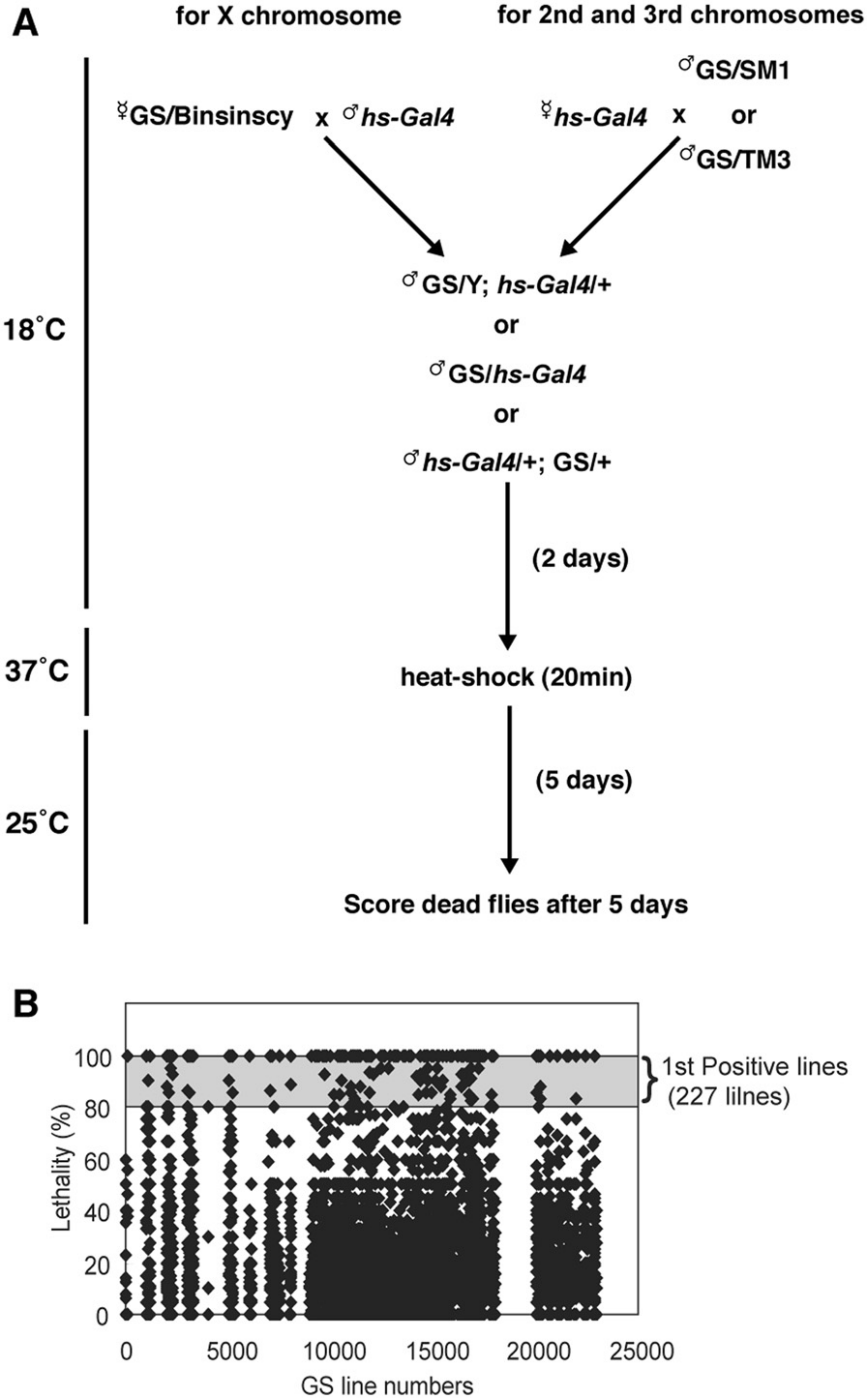
**A gain-of-function screen to identify reduced-lifespan genes. (A)** Crosses for the primary screen: GS lines were crossed with *hs-GAL4*, and their F1 progeny were raised, at 18°. Typically, 10 to 20 F1 adult males carrying each GS vector and *hs-GAL4* were collected for 5 days after eclosion, reared for another 2 days at 18°, and then heat-shocked at 37° for 20 min. The flies were reared for another 5 days at 25°, and then dead flies were counted. Lethality was calculated as the percentage of total heat-shocked flies that were dead flies. **(B)** A dot plot showing the lethality (determined by the percentage of survivors 5 days after heat shock) of individual lines; the shaded area in the graph corresponds to 80-100% lethality. GS lines with lethality greater than 80% (227 lines, indicated as 1^st^ positive lines) were used for a secondary screen.

To misexpress arbitrary genes specifically in adult flies, we maintained GS vector-bearing flies at 18° until eclosion to suppress *hs-GAL4* expression during development, and then induced *hs-GAL4* in the adult flies by heat-shocking at 37˚C for 20 min (Figure 1A), thus inducing the GAL4-driven misexpression of specific genes (Figure 1A). However, leaky expression from some of these GS lines could not be excluded under these conditions.

**Figure 2:**
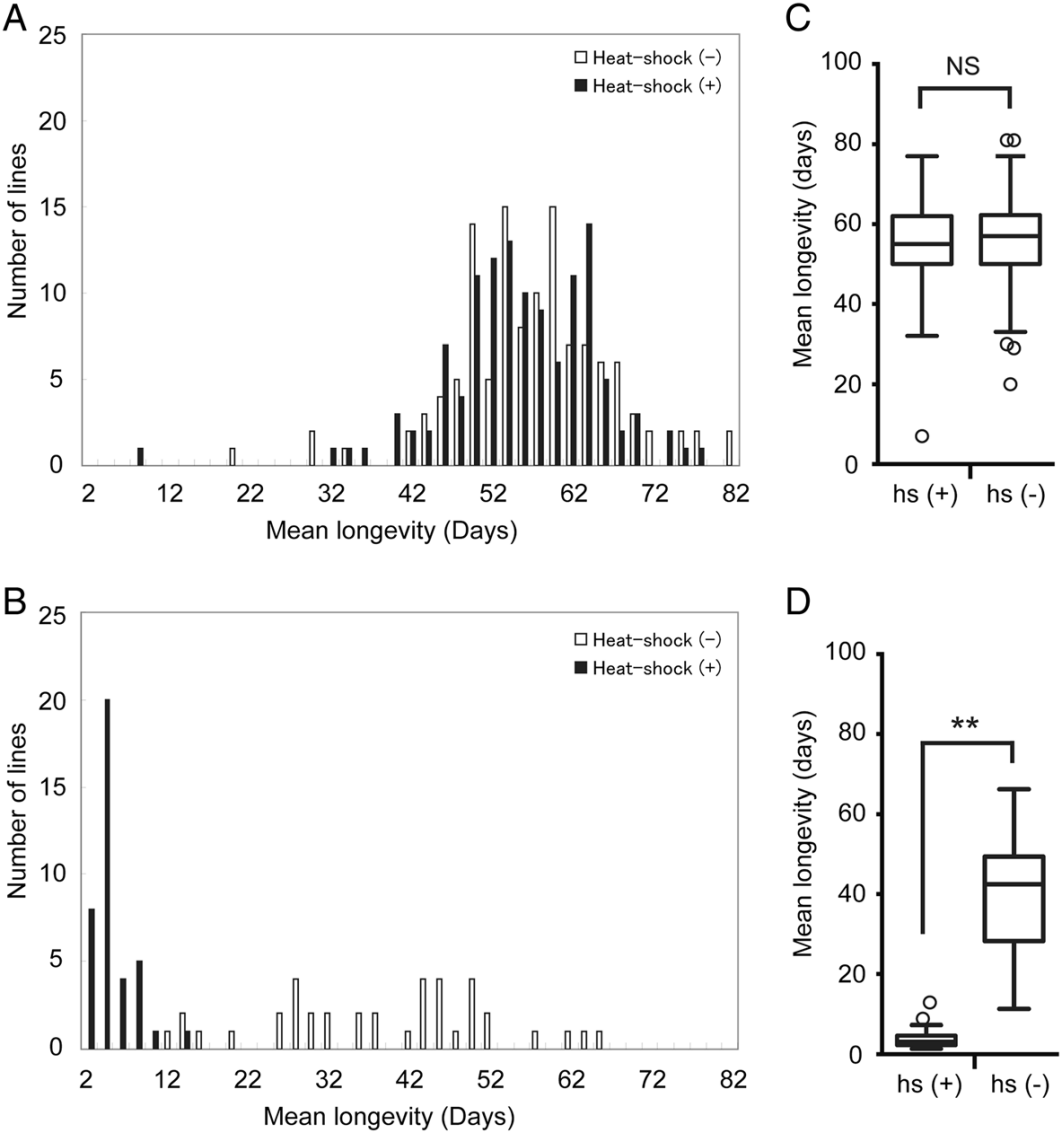
**Comparison of the mean longevity of F1 progeny with or without heat shock. (A and B)** The mean longevity of F1 progeny carrying each GS vector and *hs-GAL4* was measured with or without heat shock. The numbers of GS lines with the indicated mean longevity are shown for each 2-day period. White and black bars correspond to the number of GS lines treated without (−) or with (+) heat shock, respectively. **(A)** Mean longevity averages for 122 randomly selected GS lines: the average mean longevity of flies treated without or with heat-shock was 56.6 and 55.2 days, respectively. **(B)** GS lines misexpressing reduced-lifespan genes, identified from the screen. The average of the mean longevities of flies that were or were not heat-shocked was 3.7 and 38.5 days, respectively. **(C and D)** Box-and-whisker plots of the mean longevity averages for 122 randomly selected GS lines **(C)** and lines with reduced-lifespan genes **(D)**, with (+) or without (−) heat shock (hs). Whiskers and outliers were determined by Tukey’s method. Significance indicated (**) is P < 0.001 (unpaired t-test). NS: not significant.

Before beginning a large-scale screening of GS lines, we tested the effects of heat-shock on the longevity of adult flies to distinguish them from the effects of gene misexpression. We randomly picked up 122 GS lines and crossed them with *hs*-GAL4 flies. For each line, we collected between 20 and 40 F1 males and divided them into two groups. One group was heat-shocked at 37° for 20 min at 2 to 7 days after eclosion, and then maintained at 25°. The other group was kept at 25° without heat-shock treatment. The mean lifespan of the adult flies with or without heat-shock treatment was 55.2 ± 0.9 and 56.6 ± 0.9 days (±denotes SEM), respectively (Figure 2A); we could not find a significant difference between the groups using the t-test (Figure 2C), and the difference between the two groups was very small or negligible (the 95% confidence interval for the difference was −3.9 to 1.0 days). Therefore, the heat-shock treatment used in this study had no general effect on the longevity of the adult flies.

Using the heat-shock treatment described above, we next conducted a genome-wide screen to identify genes that severely reduced longevity when misexpressed in adulthood. As the primary screen, we selected GS lines in which more than 80% of the individual adult flies died within 5 days after misexpression of the genes. In this screen, newly eclosed F1 males were collected for 5 days at 18°, heat-shocked at 37° for 20 min, and then cultured for another 5 days at 25°; the 5-day period corresponded to less than 10% of the mean longevity of control GS flies (55.2 ± 0.9 days) under these conditions (Figure 2A).

We crossed 14,133 GS lines with *hs*-GAL4 flies. We recovered about ten F1 males from each of 12,768 GS lines, although for a few of these lines, tests were conducted using fewer flies (at least four) because of the difficulty of obtaining F1 adults in these lines. We could not recover F1 flies from crosses involving 1,365 of the GS lines. In these cases, we speculate that leaky *hs*-GAL4 expression may have driven the GAL4-dependent expression of the GS vector-regulated gene even at 18°, causing lethal developmental defects. Some of these genes might strongly affect biological processes related to cell physiology and function. However, because it could be difficult to distinguish quickly whether lethality was due to developmental defects such as cell-specification and developmental pattern formation, or due to general defects in cell physiology and functions, which we were interested in, we did not study these genes further. In total, we screened 12,768 GS lines. The genes expressed in each GS line were predicted based on the GS vector insertion site (DGSP, http://gsdb.biol.se.tmu.ac.jp/~dclust/).

In Figure 1B, the lethality of individual lines, determined by the percentage of survivors 5 days after heat shock, is indicated by a dot plot. Gene misexpression in 12,541 of the GS lines screened (98.2% of the total) did not severely reduce adult longevity; in these lines, more than 80% of the individual flies were still alive 5 days after heat-shock treatment (Figure 1B). However, in 227 of the GS lines we screened (1.8% of the total), the lifespan was significantly reduced after heat-shock treatment; less than 20% of the individual flies were still alive 5 days after heat-shock treatment (Figure 1B). In this screen, some genes were repeatedly identified in independent GS lines. For example, we identified eight independent GS lines misexpressing *Heterogeneous nuclear ribonucleoprotein at 27C*. In total, 23 genes were identified twice or more times, although six of these were excluded by the secondary screening (see below). These results suggest that acutely reduced longevity is a specific phenotype caused by the misexpression of a small number of genes, although the underlying mechanisms of these phenomena might differ between cases.

### Secondary screen

In the primary screen, we selected GS lines based on lethality measured 5 days after the heat-shock treatment. However, it was possible that this lethality was caused by developmental defects associated with leaky gene expression before adulthood. To determine whether the lethality was due to gene misexpression in the adult stage, we next compared the mean longevity of heat-shocked flies versus those not subjected to heat-shock within individual lines.

Our secondary screen analyzed 197 of the 227 positive GS lines identified in the primary screen (the remaining lines were no longer available). These GS lines were crossed with *hs*-GAL4 lines as in the primary screen, 10 to 20 F1 males were collected for each lifespan measurement, and the mean longevity of each population was measured with or without heat-shock treatment. We compared the lifespan of these adult flies with or without heat shock, and we selected GS lines in which heat shock reduced the longevity to less than 30% of that in the same line without heat shock. Based on this criterion, we obtained 47 GS lines in which heat shock significantly reduced the mean lifespan (Additional file 1: Table S1). Lines that were selected in the primary screen but did not meet the more stringent criteria of the second screen were considered GS negative. These lines may have shown a loss of longevity in the primary screen due to leaky gene expression.

The 47 positive GS lines identified in the second screen were found to drive 39 reduced-lifespan genes (see Table 1) that, when misexpressed in adulthood through heat shock, reduced the longevity to less than 30% of that of flies not subjected to heat shock (control). Figure 2B shows the mean lifespan with or without heat-shock treatment for selected positive GS lines, each expressing a reduced-lifespan gene (see Table 1). Box-and-whisker plots showed a striking difference in survival between the untreated and heat-shocked groups of these positive GS lines (Figure 2D). For example, *Transportin* misexpression in adult flies reduced their mean longevity to 3% of that of the control flies (Table 1). About half of the reduced-lifespan genes identified in this study have human orthologs that have been linked directly or indirectly to human disease (Table 2).

**Table 1:** Summary of reduced-lifespan genes

**Reduced-lifespan gene**	**GS number of 1st positive lines**	**HS/no-HS**^***c***^	**p35/GFP**^***d***^
*Transportin*	11030^*a*^, 13069^*b*^	1.9/63.0 (3.02%)	3.0/4.1 (−)
*Dream*	16231^*a, b*^	1.3/34.6 (3.76%)	41.6/1.0 (+)
*CG10277*	11124^*a, b*^, 15951	1.9/50.0 (3.80%)	1.0/2.0 (−)
*Hepatocyte nuclear factor 4*	10535^*a, b*^	1.9/48.6 (3.91%)	2.9/41.3 (−)
*Calpain-A*	11846^*a, b*^	2.1/46.0 (4.57%)	ND
*Transfer RNA:ile:49Fb*	14941^*a, b*^	3.0/65.1 (4.61%)	54.9/2.0 (+)
*Combgap*	11121^*a, b*^, 14406	2.0/43.0 (4.65%)	2.0/3.0 (−)
*trpγ*	16894^*a, b*^	1.8/36.8 (4.89%)	1.1/1.0 (−)
*Apontic*	15217^*a, b*^	2.2/44.8 (4.91%)	3.0/3.0 (−)
*Cadmus*	14710^*a, b*^	3.0/61.0 (4.92%)	2.7/3.8 (−)
*PolyA-binding protein*	13841, 15168^*a, b*^	2.4/46.7 (5.14%)	21.3/5.2 (+)
*Embryonic lethal abnormal vision*	5211^*a, b*^	2.6/43.6 (5.96%)	21.7/4.1 (+)
*CG3363*	11052^*a, b*^, 11427, 13243	3.0/49.0 (6.12%)	8.4/7.0 (−)
*Stonewall*	8015^*a, b*^, 16462, 17037	3.2/52.0 (6.15%)	6.8/21.2 (−)
*Degringolade*	3250, 15270^*a, b*^	3.0/48.6 (6.17%)	11.9/4.1 (−)
*Atlastin*	7264, 11283, 17467^*a*^	2.5/36.0 (6.94%)	2.0/1.7 (−)
*Chromator*	8043, 16669^*a, b*^, 17953	2.0/27.8 (7.19%)	3.3/3.1 (−)
*Slipper*	7470^*a, b*^	1.3/16.0 (8.13%)	31.9/1.0 (+)
*Polo*	16634^*a, b*^, 16673, 20105	3.9/44.8 (8.71%)	13.9/28.0 (−)
*Without children*	10711^*a, b*^	2.8/32.0 (8.75%)	8.1/2.1 (+)
*Heterogeneous nuclear ribonucleoprotein at 27C*	10478, 11696, 13072^*a, b*^, 13193, 14409, 14755, 16784, 16954	3.4/38.0 (8.95%)	22.3/36.5 (−)
*High mobility group protein D*	9106, 9272, 9689^*b*^, 10504, 10665^*a*^, 12910, 17197	2.6/25.0 (10.40%)	3.0/2.0 (−)
*Fmr1*	11128^*b*^, 11947^*a*^	5.0/45.0 (11.11%)	11.4/5.4 (−)
*Peroxin 16*	12435^*a, b*^, 14333, 15386	3.6/31.0 (11.61%)	17.4/22.1 (−)
*Pipsqueak*	12449^*a, b*^	6.7/51.1 (13.11%)	9.9/41.1 (−)
*CG8290*	12665^*a, b*^	4.7/34.9 (13.47%)	34.8/7.8 (+)
*Vacuolar H + ATPase subunit 100-2*	10911^*a, b*^, 14367	7.1/50.4 (14.09%)	2.8/31.6 (−)
*CG10321*	10668^*a, b*^	3.9/27.0 (14.44%)	3.2/4.4 (−)
*Roadkill*	14361, 14844, 15232, 15233^*a, b*^	6.3/41.8 (15.07%)	3.0/6.6 (−)
*Lamin*	16890^*a, b*^	4.5/28.3 (15.90%)	3.9/3.4 (−)
*Elfless*	15946^*a, b*^	3.3/20.0 (16.50%)	4.0/9.0 (−)
*CG16779*	9601^*a, b*^	4.4/26.0 (16.92%)	36.5/32.5 (−)
*Elongation factor Tu mitochondrial*	11862^*a, b*^	8.6/42.2 (20.38%)	14.9/20.1 (−)
*CG11819*	5065^*a, b*^	6.1/29.0 (21.03%)	22.7/14.8 (−)
*Autophagy-specific gene 1*	15847^*a, b*^	12.6/57.8 (21.80%)	21.8/5.0 (+)
*CG8032*	5196^*a, b*^	2.8/12.1 (23.14%)	46.9/8.3 (+)
*N-methyl-D-aspartate receptor-associated protein*	16440^*a, b*^	3.1/13.3 (23.31%)	2.8/17.7 (−)
*Src oncogene at 42A*	11022, 11049^*a, b*^	2.6/11.0 (23.64%)	4.5/5.4 (−)
*CG30482*	9799^*a, b*^	6.8/26.3 (25.86%)	5.0/28.5 (−)
Average		4.0/38.5 (10.40%)	13.5/11.6

^*a*^Representative GS lines used for second screen. The mean longevities are listed on the third column (HS/no-HS).

^*b*^GS lines used for p35 assay.

^*c*^Mean longevity (days) of heat-shocked (HS)/not-heat-shocked flies (no-HS).

^*d*^Mean longevity (days) of flies coexpressing p35 (p35)/or GFP (GFP). (+) indicates longevity extended more than three times by p35 as compared to GFP coexpression.

**Table 2:** Disease-related reduced-lifespan genes

**Reduced-lifespan gene**	**Human ortholog**	**Homology (%)**^***a***^	**Related diseases**	**Reference**
*dream*	*CASP9*	28	Alzheimer’s disease, Cancer	ROHN and HEAD [23], DEVARAJAN et al. [24]
*Hepatocyte nuclear factor 4*	*HNF4alpha*	67	Diabetes	OMIM *600281
*Calpain-A*	*CAPN3*	39	Muscular dystrophy	OMIM *114240
*embryonic lethal abnormal vision*	*ELAVL4*	53	Cancer, Anti-Hu syndrome	SZABO et al. [25]
*degringolade*	*RNF4*	42	Cancer	HIRVONEN-SANTTI et al. [26], PERO et al. [27]
*atlastin*	*ATL1*	56	Spastic paraplegia	OMIM *606439
*slipper*	*MLK2*	50	Huntington’s disease	PHELAN et al. [28]
*polo*	*PLK1*	52	Cancer	STREBHARDT [29]
*High mobility group protein D*	*SSRP1*	52	Cancer	HUDSON et al. [30]
*Fmr1*	*FXR1*	49	Fragile X mental retardation	VERKERK et al. [31]
*Peroxin 16*	*PEX16*	36	Zellweger syndrome	OMIM *603360
*Vacuolar H + ATPase subunit 100-2*	*ATP6V0A4*	51	Distal renal tubular acidosis	OMIM *605239
*roadkill*	*SPOP*	79	Cancer	LIU et al. [32]
*CG8290*	*ATRX*	36	ATRX (Xlinked αthalassemia with mental retardation)	OMIM *300032
*Lamin*	*LMNA*	38	More than a dozen different inherited diseases, including progeria syndrome	OMIM *150330
*Elongation factor Tu mitochondrial*	*TUFM*	66	Combined oxidative phosphorylation deficiency	OMIM *602389
*CG11819*	*UNC13D*	23	Familial hemophagocytic lymphohistiocytosis-3	OMIM *608897
*CG8032*	*SMOX*	33	Cancer	BABBAR and CASERO [33], GOODWIN et al. [34], XU et al. [35]
*N-methyl-D-aspartate receptor-associated protein*	*GRINA*	46	Epilepsy	BONAGLIA et al. [36]
*Src oncogene at 42A*	*FRK*	61	Cancer	HOSOYA et al. [37]

^*a*^amino acid identity (%) between reduced-lifespan genes and their human orthologs.

The misexpression of *Drosophila* genes using the GAL/UAS system is well established [22]. In addition, we here used semi-quantitative RT-PCR to confirm the misexpression of genes from GS insertions upon heat shock; the reduced-lifespan genes were markedly induced in 6 out of 8 arbitrarily selected positive GS lines (Additional file 2: Figure S1).

### Reduced-lifespan gene misexpression may reduce longevity through apoptosis

We next studied how the misexpression of these reduced-lifespan genes in adulthood reduced the longevity of adult flies. We presumed that two possibilities might account for the observed reduction in lifespan: misexpressed reduced-lifespan genes might induce ectopic cell death, leading directly or indirectly to the individual’s death, or they might cause fatal organ failure. To distinguish between these two possibilities, we studied the involvement of apoptosis in the reduction in lifespan, since several genes known to induce apoptosis were identified among the reduced-lifespan genes (Table 1). For example, *dream* is a Caspase initiator in *Drosophila*, and its misexpression induces apoptosis *in vivo*[38]. The human *slipper* ortholog *MLK2* is known to induce apoptosis in HN33 cells [39]. Thus, it was possible that some reduced-lifespan genes would be found to induce apoptosis.

To investigate this possibility, we coexpressed *p35*, which encodes a viral Caspase inhibitor, with each reduced-lifespan gene and looked for a rescue of the reduction in adult longevity seen with misexpression of reduced-lifespan genes [40]. For this experiment, the flies carried two UAS promoters, the GS and *UAS-p35* promoters. Since these promoters compete to bind GAL4 proteins, the presence of the *UAS-p35* promoter attenuates the activation of the GS promoter. To evaluate the extent to which such attenuation affected longevity, we coexpressed *UAS-GFP* as a control with each reduced-lifespan gene. The average of the mean longevities of adult flies coexpressing *UAS-GFP* and a reduced-lifespan gene was 11.6 days (Table 1). However, the average of the mean longevities of adult flies misexpressing a reduced-lifespan gene alone was 4.0 days. These results suggested that the competition of the two UAS promoters for GAL4 protein binding attenuated the expression of the reduced-lifespan genes.

**Figure 3:**
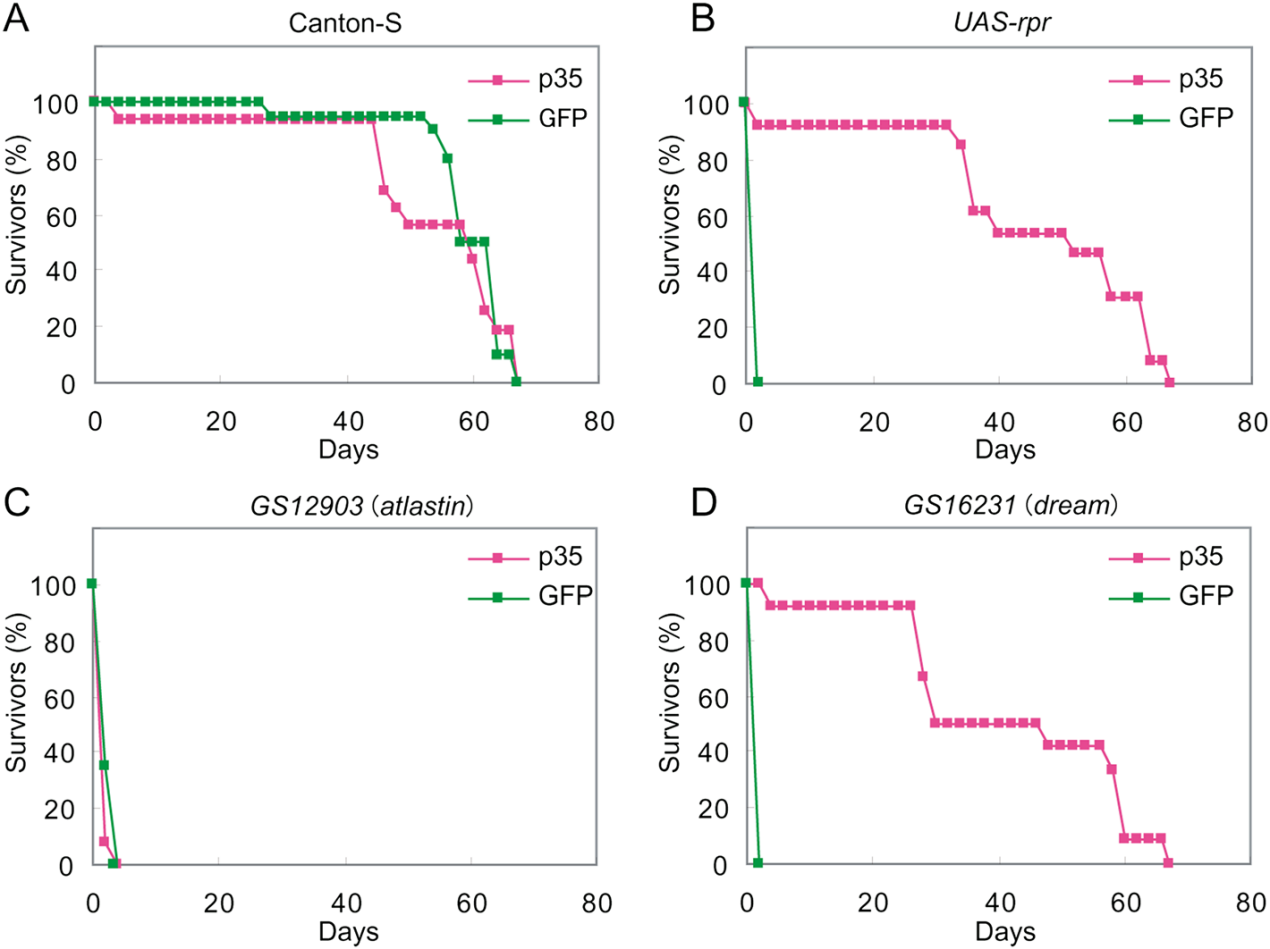
**Inhibiting apoptosis suppressed the reduced lifespan associated with misexpressed reduced-lifespan genes.** Survival curves of adult flies expressing *UAS-p35* (magenta), which encodes an apoptosis inhibitor, or *UAS-GFP* (green). Survivor rates are shown for 2-day intervals. **(A)** Control (Canton-S) flies; **(B-D)** flies coexpressing the reduced-lifespan gene **(B) ***UAS-rpr*, **(C) ***atlastin* (misexpression driven by *GS12903*), or **(D) ***dream* (misexpression driven by *GS16231*).

The effect of this attenuation differed among the reduced-lifespan genes. For 10 of the 39 reduced-lifespan genes, coexpressing *UAS-GFP* with the misexpressed gene more than tripled the mean longevity of the adult flies (Table 1). However, for the other 29 reduced-lifespan genes, coexpressing *UAS-GFP* did not significantly extend the adults’ mean longevity. These results suggest that the threshold level at which the gene expression reduces the adult lifespan differs among the reduced-lifespan genes. The mean longevity of flies that misexpressed either *UAS-p35* or *UAS-GFP,* but were otherwise wild-type, was 53.3 and 59.3 days, respectively (Figure 3A). Therefore, misexpressed *UAS-p35* or *UAS-GFP* alone did not affect the longevity of adult flies.

Before examining the effect of p35 on the individual reduced-lifespan genes identified in our screen (Table 1), we performed another control experiment in which we tested whether the reduced life span induced by apoptosis could be restored by misexpressing *UAS-p35*. In *Drosophila*, the *rpr* gene is required for the induction of apoptosis. Ectopic *rpr* expression induces apoptosis *in vivo*[41], and *rpr*-induced apoptosis is effectively suppressed by p35 coexpression *in vivo*[42]. Here, we found that the misexpression of *rpr* in adulthood severely reduced adult longevity (to 1.3 days) (Figure 3B); this shortened longevity was significantly restored by coexpressing *UAS-p35* (Figure 3B). These results suggested that p35 could effectively suppress the reduction in lifespan caused by ectopically induced apoptosis in adulthood.

We next examined how the reduction in lifespan caused by misexpressed reduced-lifespan genes in adults was affected by coexpressing *UAS-p35*. Each reduced-lifespan gene was coexpressed with either *UAS-p35* or *UAS-GFP,* driven by heat-shock, in adult flies, and the mean longevity of the two populations was measured (Table 1). We found that the longevity shortened by the misexpression of nine of the 39 reduced-lifespan genes was increased over three times in flies coexpressing *UAS-p35* compared with those coexpressing *UAS-GFP* (Table 1), suggesting that these nine reduced-lifespan genes directly or indirectly induced apoptosis, severely shortening the adult lifespan. Survivor rates, measured every three days, were presented as survival curves of the adult flies coexpressing each reduced-lifespan gene and either *UAS-p35* or *UAS-GFP* (Figure 3 and Additional file 3: Figure S2). As a control, *UAS-rpr* was coexpressed with *UAS-p35* or *UAS-GFP* (Figure 3B). The survivor curves associated with the misexpression of these nine reduced-lifespan genes shared similarities with the *UAS-rpr* curves. For example, as with *UAS-rpr*, the survivor curves involving *dream* indicated that the reduced lifespan was effectively suppressed by the coexpression of *UAS-p35* but not of *UAS-GFP* (Figure 3D). These results suggested that apoptosis plays a critical role in the severe reduction in longevity when one of these nine genes is misexpressed in adults.

To confirm this idea, we examined the ectopic apoptosis induced by the adult-specific expression of these nine genes, using TUNEL assays [43]. In adult flies, the brain is one of the most suitable organs for observing ectopic apoptosis. For example, apoptotic neurodegeneration is labeled by TUNEL in the adult brain in a *Drosophila* model of neurodegenerative disease [44]. As a control, *UAS-rpr* misexpression in adults was driven by *hs-GAL4*, as performed in our screens. The brain was dissected out from flies that were still alive after more than 50% of the adult flies had died, and the apoptosis in the brain was assayed by TUNEL (Additional file 4: Figure S3, B). Under these conditions, the adult-specific misexpression of *dream*, *Autophagy-specific gene 1*, and *CG8032* ectopically induced apoptosis in the brain (Additional file 4: Figure S3, C-E). Based on the percentage of TUNEL-positive brains upon the adult-specific misexpression of these nine reduced-lifespan genes, which probably reduce adult longevity through apoptosis, our results suggest that *dream*, *Autophagy-specific gene 1*, and *CG8032* are more potent apoptosis inducers than the other six reduced-lifespan genes tested (Additional file 4: Figure S3, F).

On the other hand, the survivor curves associated with other reduced-lifespan genes, for example, *atlastin* (*atl*), when coexpressed with either *UAS-p35* or *UAS-GFP* were largely comparable (Figure 3C, and Table 1), suggesting that the shortened longevity associated with the misexpression of this class of reduced-lifespan genes may not be accounted for by the induction of apoptosis—although it is possible that these genes induce apoptosis that is not suppressed by *p35* coexpression.

### Tissues responsible for shortening longevity when reduced-lifespan genes are misexpressed

In our analysis above, the effect of reduced-lifespan genes on longevity was evaluated by their misexpression in the whole body of the adult fly. To better understand how each of these genes causes reduced lifespan, it is important to determine which tissues or organs are responsible for the reduced lifespan when the gene is misexpressed. To examine this, we controlled the reduced-lifespan gene expression temporally and spatially using the TARGET system [45], which combines temperature-sensitive GAL80 (GAL80^ts^) control of GAL4 activity with a GAL4/UAS system. The *Drosophila tub-GAL80*^*ts*^ line ubiquitously expresses *GAL80*^*ts*^*in vivo*[45]. Tissue-specific GAL4 expression was driven by various GAL4 driver lines. In flies carrying a GAL4 driver and *tub-GAL80*^*ts*^, the GAL4 activity is suppressed at 19° and restored at 30° [45].

We used eight different GAL4 driver lines to express GAL4 in specific tissues. The GAL4 expression pattern was confirmed in the adult tissues of each of these lines by crossing them with the *UAS-GFP* line (Table 3). To analyze the *GAL4* expression patterns in the brain and gut in more detail, we stained these tissues with an anti-GFP antibody. The results confirmed that these GAL4 lines showed representative differential *GAL4* expression (Additional file 5: Figure S4).

**Table 3:** Expression patterns of GAL4 drivers in adults

**GAL4 driver**	**Expression pattern in adult tissues**
*P{GawB}l(3)31-1*^*31–1*^	Brain, basiconical sensilla, thoracico-abdominal ganglion, salivary glands
*48Y-GAL4*	Brain, anterior-midgut, paragonia, salivary glands
*24B-GAL4*	Somatic musculature, visceral musculature, salivary glands
*NP5021*	Whole gut
*byn-GAL4*	Hindgut
*drm-GAL4*	Small intestine
*Cg-GAL4*	Hemocytes
*P{GawB}5108*	Salivary glands

**Figure 4:**
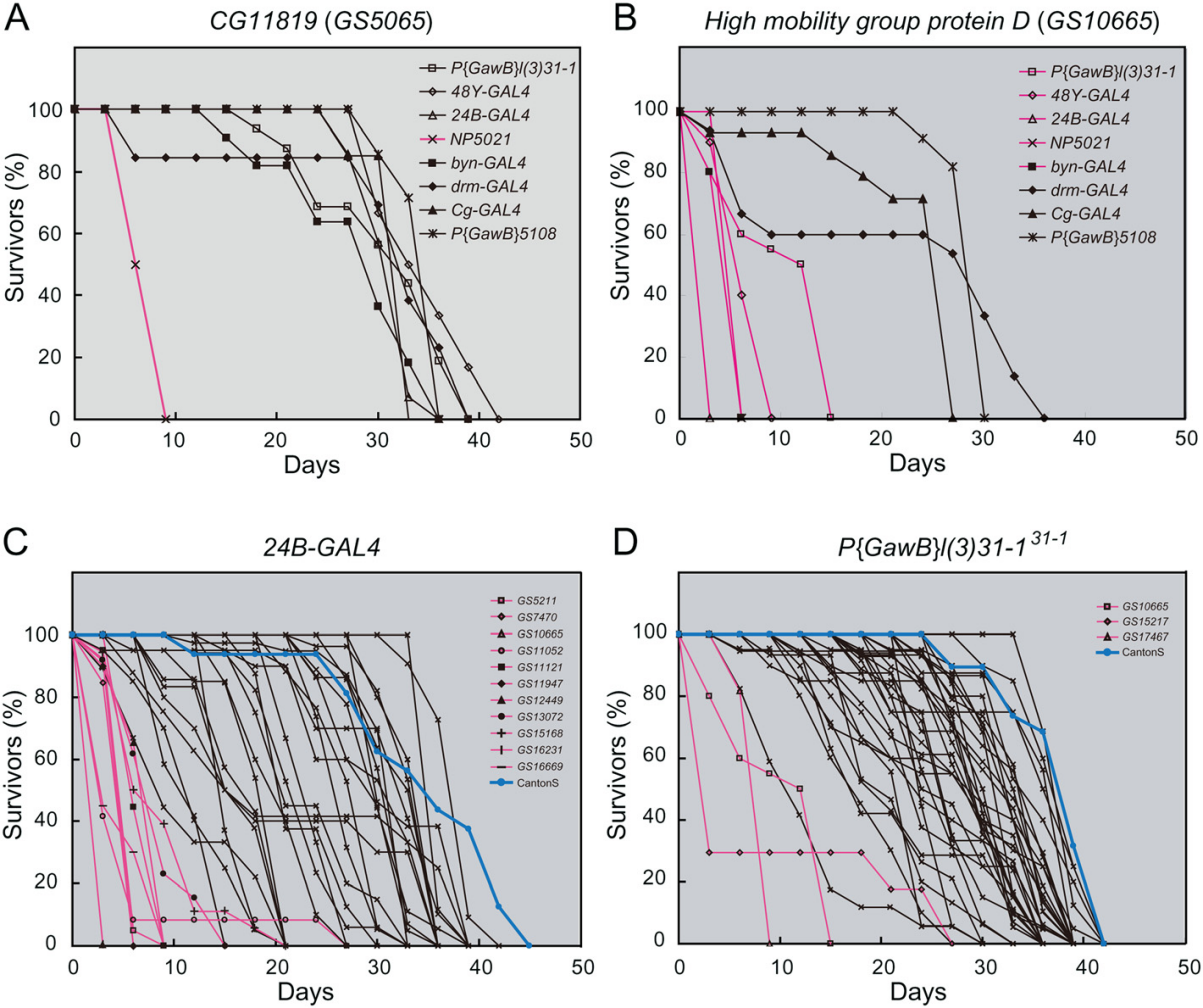
**Misexpression of reduced-lifespan genes in specific tissues was responsible for reduced lifespan in adult flies.** Misexpression of reduced-lifespan genes in specific tissues was responsible for reduced lifespan in adult flies. **(A-D)** Survival curves of adult flies misexpressing reduced-lifespan genes. Tissue-specific expression of reduced-lifespan genes in adult flies was induced with the TARGET system. Survivor rates were scored every three days. **(A, B)** Two representative reduced-lifespan genes (A) *GS5065* (*CG11819*) and **(B) ***GS10665* (*High mobility group protein D*) were misexpressed using various GAL4 drivers, indicated in the upper right. **(C, D)** Survival curves for all identified reduced-lifespan genes when driven by the GAL4 driver **(C) ***24B-GAL4* or (D) *P{GawB}l(3)31-1*^*31–1*^. Blue lines: survival curve of a wild-type (Canton-S) control. Magenta lines: survival curves showing a lifespan less than 1/3 that of wild-type control flies; the corresponding reduced-lifespan genes are shown in the upper right. Survival curves showing a lifespan greater than 1/3 of that of wild-type control flies are shown in black; the corresponding gene names are not shown.

To suppress the expression of reduced-lifespan genes until adulthood, flies carrying various GAL4 drivers, *tub-GAL80*^*ts*^, and GS vectors were cultured at 18° until eclosion. Adult flies were collected for 5 days, and 2 days later the misexpression of reduced-lifespan genes in specific adult tissues was induced by a temperature shift from 18° to 30°. We measured the survivor rate every three days and calculated the mean longevity (Table 4). In these experiments, longevity was measured in flies maintained at 30°, which generally have a shorter lifespan than those cultured at 25° (Table 1; Table 4). Therefore, we compared their longevity with that of otherwise wild-type flies expressing GAL4 (Canton-S in Table 4) at 30°. We successfully identified reduced-lifespan genes that, when expressed in specific tissues, reduced the mean longevity to less than one-third of that in control flies (Table 4 and magenta in Figure 4).

**Table 4:** Mean longevity of adult flies with the misexpression of reduced-lifespan genes in specific adult tissues

**Reduced-lifespan gene**	**GS line. no.**	**GAL4 driver line**							
		***P{GawB}l(3)31-1***^***31–1***^	***48Y-GAL4***	***24B-GAL4***	***NP5021***	***byn-GAL4***	***drm-GAL4***	***Cg-GAL4***	***P{GawB}5108***
*Transportin*	11030	33.4	33.5	32.7	15.6	39.8	33.5	25.6	33.0
*Dream*	16231	29.1	34.7	**3.0**	**4.6**	21.8	25.2	30.9	(30.0)
*CG10277*	11124	35.4	32.5	23.4	**7.6**	25.1	27.9	27.9	32.1
*Hepatocyte nuclear factor 4*	10535	37.8	29.1	27.7	13.8	25.3	30.2	31.6	35.5
*Calpain-A*	9176	30.0	(37.8)	23.5	17.8	(19.5)	21.3	23.5	29.4
*Transfer RNA:ile:49Fb*	14941	33.3	(19.2)	33.7	**4.1**	28.8	31.2	29.0	36.8
*Combgap*	11121	27.6	28.8	**5.5**	20.1	**7.8**	20.5	30.7	(28.8)
*trpγ*	16894	33.2	31.0	31.0	19.2	30.8	27.0	35.3	(31.2)
*Apontic*	15217	**9.4**	27.9	36.3	**7.8**	34.4	29.1	31.7	25.5
*Cadmus*	14710	33.3	35.3	38.2	26.7	36.9	21.0	26.6	34.3
*PolyA-binding protein*	15168	33.0	32.1	**9.3**	**5.3**	23.2	29.8	26.3	35.1
*Embryonic lethal abnormal vision*	5211	27.7	31.1	**6.0**	30.0	12.2	33.0	26.6	31.5
*CG3363*	11052	20.7	18.5	**8.8**	11.6	22.4	19.5	21.0	33.9
*Stonewall*	8015	24.8	33.4	12.6	**10.2**	18.7	32.6	17.1	36.0
*Degringolade*	15270	22.6	33.3	20.1	**9.2**	17.0	11.6	29.7	36.5
*Atlastin*	17467	**8.5**	35.1	27.3	16.2	17.3	31.7	30.2	42.2
*Chromator*	16669	25.5	17.1	**5.3**	13.1	**9.6**	**9.6**	14.6	(27.0)
*Slipper*	7470	13.1	14.8	**7.3**	26.8	21.9	25.5	**8.1**	26.7
*Polo*	16634	30.9	13.6	29.6	24.3	24.9	35.1	28.8	35.6
*Without children*	10711	27.3	38.1	16.3	**8.1**	22.3	26.3	16.7	29.0
*Heterogeneous nuclear ribonucleoprotein at 27C*	13072	18.5	13.8	**6.0**	13.8	**5.1**	15.6	25.8	33.5
*High mobility group protein D*	10665	**10.4**	**6.9**	**3.0**	**6.0**	**5.4**	21.6	23.4	29.2
*Fmr1*	11947	30.0	21.4	**5.8**	14.8	15.0	23.7	14.5	(36.6)
*Peroxin 16*	12435	34.3	32.0	24.0	12.8	32.4	18.6	31.7	(21.6)
*Pipsqueak*	12449	27.3	30.4	**7.7**	**6.0**	25.4	30.5	29.4	29.0
*CG8290*	12665	32.1	21.7	19.2	26.4	16.4	33.6	32.4	37.0
*Vacuolar H + ATPase subunit 100-2*	10911	35.8	(35.4)	34.2	26.4	24.4	29.1	35.3	(37.2)
*CG10321*	10668	24.6	35.0	12.0	**10.5**	13.4	35.3	19.3	32.8
*Roadkill*	15233	32.9	38.5	27.9	31.1	34.1	32.8	31.4	(35.3)
*Lamin*	16890	21.5	22.5	21.6	**6.0**	12.2	17.8	10.9	23.9
*Elfless*	15946	30.6	28.4	22.8	20.6	20.6	31.4	29.9	34.4
*CG16779*	9601	26.9	34.5	11.7	16.5	31.2	34.5	18.0	45.0
*Elongation factor Tu mitochondrial*	11862	29.0	28.3	23.3	22.8	31.5	15.5	31.3	(27.6)
*CG11819*	5065	31.1	35.0	31.5	**7.5**	28.1	30.2	32.1	34.7
*Autophagy-specific gene 1*	15847	29.5	34.2	32.6	14.7	28.1	27.2	31.7	37.8
*CG8032*	5196	38.4	28.4	33.9	12.6	30.0	35.1	28.8	32.0
*N-methyl-D-aspartate receptor-associated protein*	16440	27.6	**5.5**	15.4	15.8	20.6	16.7	23.9	31.5
*Src oncogene at 42A*	11049	21.6	21.9	17.1	**9.8**	19.2	24.6	16.1	33.0
*CG30482*	9799	32.8	37.4	30.8	31.1	29.3	**9.9**	13.8	35.8
Canton-S (control)		37.6	32.9	34.9	31.5	31.8	35.7	28.4	34.4

Bold numbers indicate longevity less than 1/3 of control. Parenthetical numbers indicate the mean longevity obtained from fewer than five flies.

Of the 39 reduced-lifespan genes identified in our screens, we found 24 that reduced the adult longevity when their misexpression was driven by one or more GAL4 drivers specifically in adulthood. Among these, 15 genes shortened longevity only when their misexpression was driven by one particular GAL4 driver (Figure 4A, and Additional file 6: Figure S5). On the other hand, nine reduced-lifespan genes severely reduced the adult longevity when misexpressed by at least two different GAL4 drivers (Table 4). For example, *High mobility group protein D* severely shortened longevity when its misexpression was driven by several GAL4 lines (Table 4, Figure 4B, and Additional file 6: Figure S5). However, it remains unknown whether each of these reduced-lifespan genes induced shortened longevity in two different tissues by distinct underlying mechanisms.

Susceptibility to the misexpression of reduced lifespan differed among tissues. Many reduced-lifespan genes induced shortened longevity when misexpressed under the GAL4 drivers *24B-GAL*4 or *NP5021*, but not under other GAL4 drivers (Table 4). Of the 39 reduced-lifespan genes, the misexpression of 11 shortened the lifespan when driven by *24B-GAL4* (brain, visceral muscle, and Malpighian tubules) and 14 when driven by *NP5021* (gut epithelium and Malpighian tubules) (Table 4, Figure 4C, and data not shown). These results suggest that the gut and Malpighian tubules are more susceptible to many misexpressed reduced-lifespan genes. Unexpectedly, when the misexpression was driven by *48Y-GAL4* or *P{GawB}l(3)31-1*^*31–1*^, which highly express *GAL4* in the brain (medium and strong levels in (Additional file 5: Figure S4), only two or three of the 39 reduced-lifespan genes severely reduced adult longevity (Table 4 and Figure 4D), suggesting that neuronal cells may have some tolerance to perturbation by misexpressed genes. In contrast, while the neuron-specific misexpression of *dream* driven by *48Y-GAL4* did not reduce longevity, its misexpression in the gut epithelium and Malpighian tubules severely reduced it, as mentioned above (Table 4). Since the reduction in longevity by *dream* misexpression was effectively restored by coexpressing *p35*, apoptosis in neuronal cells may not strongly influence adult longevity.

### Classification of reduced-lifespan genes by gene ontology

To explore common biological functions among the reduced-lifespan genes, we conducted gene ontology (GO) analysis. Gene ontology provides annotations for each gene along with insights into the biology of the system being studied [46]. We focused on biological process, which is one of three main GO categories. This category contains 30490 GO terms. One or more GO term was found for 10535 *Drosophila melanogaster* genes. We found that 33 of the 39 reduced-lifespan genes had at least one GO term. We then searched for significantly shared GO terms associated with selected genes. The p-value was calculated by a one-tailed Fisher’s exact test (p-value < 0.001). We filtered the GO terms containing more than 10 reduced-lifespan genes. Significant shared terms are shown in Table 5. The GO term “developmental process” had the lowest p-value of all the terms. In addition, five out of seven terms were related to development. These results indicate a bias of reduced-lifespan genes toward development-related genes.

**Table 5:** Significant shared GO terms between reduced-lifespan genes and total genes

**GO ID**	**GO ACCESSION**	**GO Term**	**p-value**	**Count in reduced-lifespan genes**	**% Count in reduced-lifespan genes**	**Count in total genes**	**% Count in total genes**
14320	GO:0032502	Developmental process	3.81E-05	16	48.5	1830	17.4
4795	GO:0007275	Multicellular organismal development	1.78E-04	14	42.4	1620	15.4
22125	GO:0048513	Organ development	3.56E-04	10	30.3	921	8.7
22457	GO:0048856	Anatomical structure development	5.87E-04	12	36.4	1375	13.1
6464	GO:0009653	Anatomical structure morphogenesis	6.05E-04	10	30.3	984	9.3
22337	GO:0048731	System development	6.12E-04	11	33.3	1178	11.2
14319	GO:0032501|GO:0050874	Multicellular organismal process	7.14E-04	15	45.5	2080	19.7

Among the 39 reduced-lifespan genes, we found that human orthologs of six genes—*embryonic lethal abnormal vision*, *Heterogeneous nuclear ribonucleoprotein at 27C*, *Vacuolar H*^*+*^*ATPase subunit 100–2*, *Elongation factor Tu mitochondrial*, *High mobility group protein D*, and *polyA-binding protein*—are known as housekeeping genes [47]. These results suggested that the overproduction of proteins encoded by some housekeeping genes could affect cell physiology, which could directly or indirectly shorten longevity.

On the other hand, only four reduced-lifespan genes had an apoptosis-related GO term. The reduced-lifespan phenotype induced by the adult-specific expression of three of these, *dream*, *polyA-binding protein*, and *Autophagy-specific gene 1,* was suppressed by coexpressing *UAS-p35* (Figure 3 and Table 1), suggesting that apoptosis was a potential cause of the reduced longevity. Thus, for these three reduced-lifespan genes, the GO analysis results were consistent with our observations using *UAS-p35*. However, *UAS-p35* coexpression suppressed the reduced-lifespan phenotype for six other reduced-lifespan genes (Table 1) that did not have an apoptosis-related GO. Therefore, we speculated that these six reduced-lifespan genes indirectly induce apoptosis by disrupting other physiological processes.

## Conclusions

In this study, we identified 39 genes that induced reduced lifespan in flies when misexpressed in adulthood. In our screen, we did not study GS lines that were lethal during embryonic, larval, and/or pupal stages due to leaky gene expression driven by GS insertion. We speculate that these genes may affect cell specification or pattern formation during development, although some may strongly influence cell physiology and function at various stages of the *Drosophila* life cycle. Our results suggest that ectopic apoptosis and/or organ malfunction are responsible for the reduced lifespan induced by the misexpression of reduced-lifespan genes in adulthood. Based on the differences in sensitivity to apoptosis inhibition and in the tissues responsible for shortened life, the causes of reduced lifespan differ among the reduced-lifespan genes.

## Methods

### *Drosophila* strains

Canton-S was used as a wild-type strain. Flies were raised at 25° unless otherwise specified. The Gene Search (GS) collection was obtained from the Drosophila Gene Search Project [16]. The UAS lines *UAS-p35*, *UAS-reaper* (*rpr*), and *UAS-GFP* were obtained from the Bloomington Stock Center. The following GAL4 drivers were used: *P{GAL4-Hsp70.PB}* (Bloomington Stock Center #2077), *P{GawB}l(3)31-1*^*31–1*^ (Bloomington Stock Center #5820), *48Y-GAL4*[48], *24B-GAL4*[49], *drumstick* (*drm*)*-GAL4*[50], *Cg-GAL4* (Bloomington Stock Center #7011), *brachyenteron* (*byn*)-*GAL4*[51], *NP5021*[52], and *P{GawB}5108* (Bloomington Stock Center #2736); *tub-GAL80*^*ts*^ was also obtained from the Bloomington Stock Center (#7016 and #7017).

### Genetic screening

GS lines express arbitrary genes downstream of the upstream activation sequence (UAS), depending on the insertion locus of each GS vector [16]. To overexpress these genes specifically in adult flies, we used a heat-inducible GAL4 driver, *P{GAL4-Hsp70.PB}*, referred to as *heat-shock-GAL4* (*hs-GAL4*) [17]. Figure 1A shows the scheme of genetic crosses performed for this screen. GS flies carrying GS vectors inserted on the 2nd or 3rd chromosome were crossed with virgin *hs-GAL4* females. For GS lines with a GS vector on the X chromosome, virgin females were collected and crossed with *hs-GAL4* males. To suppress potential leaky *hs-GAL4* expression during development, flies were crossed and the offspring (F1) raised at 18°.

For the primary screen, in most cases 10 to 20 F1 males were collected for 5 days starting from the first day of eclosion, and were kept at 18° for two days. These F1 flies (2 to 7 days old) were heat-shocked at 37° for 20 min, and then maintained at 25°. Dead flies were counted 5 days after the heat-shock treatment, and lethality was defined as the percentage of all heat-shocked flies that were dead flies.

For the second screen, in most cases 20 to 40 F1 flies from the potentially positive GS lines, obtained as described for the primary screen, were divided into equal groups. One group was heat-shocked at 25° for 20 min and the other was not heat-shocked; lethality was measured for each group as described for the primary screen. GS vector insertion sites, if not already reported, were determined by genomic PCR as described by the *Drosophila* Gene Search Project [16].

### Semi-quantitative RT-PCR

Arbitrarily selected GS lines capable of driving reduced-lifespan genes were crossed with *hs-GAL4*, and the F1 flies were cultured by the same method used in the secondary screen. Three adult flies for each experiment were heat-shocked (37˚) or non-heat-shocked (18˚) for 20 min and subsequently maintained for 6 hours at 25°. From these flies, the total RNA was extracted using Isogen (Nippon Gene), and treated with RNase-free DNase (Takara). Semi-quantitative RT-PCR was performed as described before [53]. First-strand cDNA was synthesized using the 1^st^ strand cDNA Synthesis Kit (Takara), and PCR was performed using ExTaq (Takara). The following oligonucleotides were used as PCR primers: *Transportin*, 5′-GAGGAGACCAAGCAGTACATACG-3′ and 5′-TTCAACTGGGCACACATAACC-3′; *CG10277*, 5′-GACACCTGTGTGATCTGTCTGG-3′ and 5′-GCCCTTCGTAAAAACCTTGC -3′; *Calpain-A*, 5′-GGTTCCCTTTTCGAAGATCC-3′ and 5′-GAACATCAAAACGCGAATAACC-3′; *combgap*, 5′-TCAAGCACCATTTGACAACC-3′ and 5′-GCACTCCTGGCACTGATAAGG-3′; *polyA-binding protein*, 5′-GCTGTCCATTCGTGTCTGC-3′ and 5′-CGACGAAGAGAAGGATCACG-3′; *CG3363*, 5′-CTCCCAAATGCCTTTTACCC-3′ and 5′-CTCGCGCTTCAAATTATTGC-3′; *stonewall*, 5′-CAGACTGCGCTTTATGATCG-3′ and 5′-CCAGCGGGTATAGTCATTTCG-3′; *polo*, 5′-ACATCAACCAGCGGAAAACC-3′ and 5′-TGTTTGATCATCAGCTTCTTGG-3′; *Glyceraldehyde 3 phosphate dehydrogenase 1* (*Gapdh1*), 5′-GGAGCCACCTATGACGAAATCAA-3′ and5′- GACGAATGGGTGTCGCTGAA-3′. The PCR products were analyzed by 1% agarose gel electrophoresis. *Gapdh1* was used an internal control for semi-quantification.

### Apoptosis inhibition by coexpressing *p35* with reduced-lifespan genes

The baculoviral protein p35 efficiently inhibits the apoptosis signaling cascade by competing with Caspase substrates [54]. GS lines were crossed with *UAS-p35*; *hs-GAL4* and *UAS-GFP*; *hs-GAL4*. We collected 10 to 20 F1 flies carrying a GS vector, *hs-GAL4*, and *UAS-p35* or *UAS-GFP*. The flies were heat-shocked as described above and reared at 25°. Dead flies were counted every two days. UAS-*rpr* was used for a positive control.

### TUNEL assay

Adult brains were dissected and fixed (4% paraformaldehyde in PBS) for 30 min at room temperature. The brains were washed with PBS, and the endogenous peroxidase activity was blocked by incubating the samples with 0.3% H_2_O_2_ in methanol for 30 min at room temperature. The brains were washed twice in PBS, once in 0.1% Triton X-100/0.1% sodium citrate for 20 min on ice, and then twice in PBS. The TUNEL assay was performed according to the manufacturer’s instructions (In Situ Cell Death Detection Kit, Roche). Fluorescent images were obtained by confocal microscopy, LSM700 (Carl Zeiss).

### Tissue-specific expression of reduced-lifespan genes in adulthood

The temporal and regional gene expression targeting (TARGET) system was used to express genes in specific tissues in adult flies [45]. The following GAL4 drivers were used: *P{GawB}l(3)31-1*^*31–1*^, *48Y-GAL4*, *24B-GAL4*, *NP5021*, *byn-GAL4*, *drm-GAL4*, *Cg-GAL4*, and *P{GawB}5108*. GAL4 expression in adult tissues was examined by crossing these GAL4 lines with *UAS-GFP*; the expression patterns are summarized in Table 3. GAL80^ts^, which suppresses GAL4 at 18° but not at 30° [45], was expressed from *tub-GAL80*^*ts*^ on the X chromosome (#7016) and the third chromosome (#7017). Flies carrying a GAL4 driver and *tub-GAL80*^*ts*^ were crossed with GS lines with GAL4-controlled expression of reduced-lifespan genes, and the F1 progeny of these crosses were raised at 18° to suppress gene expression regulated by the GS vector. Typically, 10 to 20 F1 males were collected within 5 days after eclosion and incubated for 2 more days at 18°. These adult flies were then cultured at 30° to drive the expression of reduced-lifespan genes in tissues expressing GAL4. Dead flies were counted every 3 days, and mean longevities calculated as described above. Canton-S (wild-type) flies were used as a negative control.

### Analysis of tissue-specific GAL4 expression driven by various GAL4 lines

To analyze the tissue-specific expression of *GAL4* in various GAL4 lines used in this study, GAL4 lines (indicated at the left in Additional file 5: Figure S4) were mated with *UAS-GFP*. The F1 adults were cultured at 25°. The brain and digestive organs were dissected out, fixed with 4% paraformaldehyde in PBS, and stained with an anti-GFP antibody (1:500 dilution, Invitrogen Life Technologies) and Alexa-Fluor secondary antibody (1:200 dilution, Invitrogen Life Technologies). The *GFP* expression was observed by confocal microscopy, LSM700 (Carl Zeiss).

### Gene ontology analysis

We used Genespring GX version 10.0 to perform gene ontology analyses and to extract the enriched gene ontology terms among the genes shortening longevity. The P-value of each enriched gene ontology term was obtained by a one-tailed Fisher’s exact test.

## Competing interests

The authors declare that they have no competing interests.

## Authors’ contributions

MN, TA, YM, ST, and KM, designed the experiments. MN, TI, HS, TU, TO, HY, HI, YT, AM, KK, MT, JK, MA, AT, TS, and TY conducted the experiments. MN, HOI, WG, and MO analyzed and interpreted the data. MN, HOI, and KM drafted the manuscript. All authors read and approved the final manuscript.

## Supplementary Material

Additional file 1: Table S1 All GS lines positive for the secondary screen. Click here for file

Additional file 2: Figure S1 Semi-quantitative RT-PCR confirmed the misexpression of reduced-lifespan genes upon heat-shock treatment. Results of semi-quantitative RT-PCR detecting cDNA fragments of *Transportin*, *CG10277*, *Calpain-A*, *combgap*, *polyA-binding protein*, *CG3363*, *stonewall*, and *polo* are shown. Total RNA was isolated from adult flies carrying *hs-GAL4* and a GS insertion that were subjected to the same culture conditions used in our secondary screen, with or without heat-shock treatment. The numbers 21, 24, 27, and 30 are PCR cycles. Heat shock markedly increased the RT-PCR products from *Transportin*, *CG10277*, *combgap*, *CG3363*, *polo*, and *stonewall. Glyceraldehyde 3 phosphate dehydrogenase 1* (*Gapdh1*) was used as an internal control. *polo* gave an additional RT-PCR product (shown by an asterisk). Click here for file

Additional file 3: Figure S2 Survival curves of adult flies expressing UAS-p35 (magenta), which encodes an apoptosis inhibitor, or UAS-GFP (green), in combination with each reduced-lifespan gene (shown at the top of each graph). Details are described in Figure 3. Click here for file

Additional file 4: Figure S3 The adult-specific misexpression of reduced-lifespan genes induced apoptosis in the adult brain. Canton-S (wild-type), *UAS-rpr*, and positive GS lines were crossed with *hs-GAL4*, and the F1 flies were heat-shocked as in the primary and secondary screens. Brains were dissected from flies that were still alive after more than half of the heat-shocked flies had died, and apoptotic cells were detected by TUNEL assay. Fluorescence microscopy images of adult brains are shown. Canton-S **(A)** and *UAS-rpr***(B)** were used as negative and positive controls, respectively. **(C-E)** Representative samples of positive GS lines, GS16231 (*dream*) **(C)**, GS15847 (*Autophagy-specific gene 1*) **(D)**, and GS5196 (*CG8032*) **(E)**, are shown. White arrowheads indicate TUNEL-positive apoptotic cells. **(F)** Frequency of TUNEL-positive brains is shown as a percentage. The number of samples tested is shown above each bar. Click here for file

Additional file 5: Figure S4 Tissue-specific expression of *GAL4* in the adult brain, gut, and Malpighian tubule in various GAL4 lines. We analyzed the *GAL4* expression patterns in six lines (indicated at left). The *UAS-GFP* expression driven by each GAL4 line was analyzed by confocal microscopy after immunostaining for the GFP protein (green) in the adult brain and in the gut /Malpighian tubule. Corresponding optical microscopy images are shown at right. Arrows indicate visceral muscles; arrowheads show the Malpighian tubule. The regions outlined by white broken lines are magnified in panels to the immediate right. The relative intensity of *GFP* expression in various GAL4 lines is indicated in the bottom table, as - (negative), +/− (almost negligible), + (weak), ++ (medium), and +++ (strong). Click here for file

Additional file 6: Figure S5 Tissue-specific expression experiments. Survival curves of adult flies misexpressing each reduced-lifespan gene. Tissue-specific expression was induced in adult flies with the TARGET system, and survivor rates were scored every three days. Details are described in Figure 4. Click here for file
