# Supplementary figures and images for: A gain-of-function screen to identify genes that reduce lifespan in the adult of Drosophila melanogaster
Source: BMC Genet. 2014 Apr 16;15:46. doi: 10.1186/1471-2156-15-46 (PMC4021436; doi:10.1186/1471-2156-15-46)

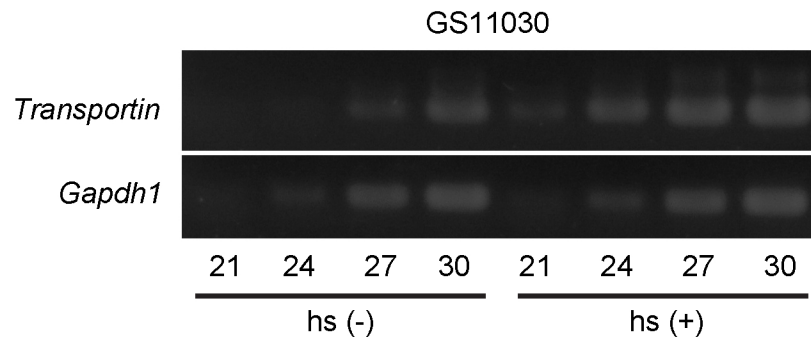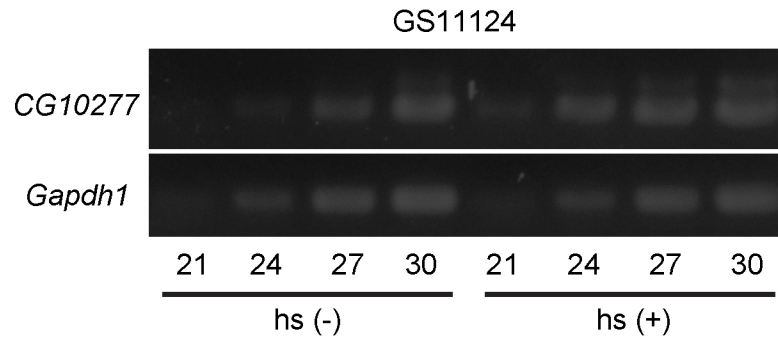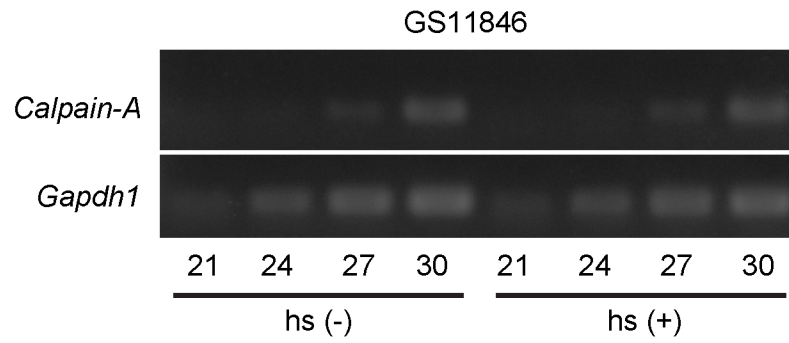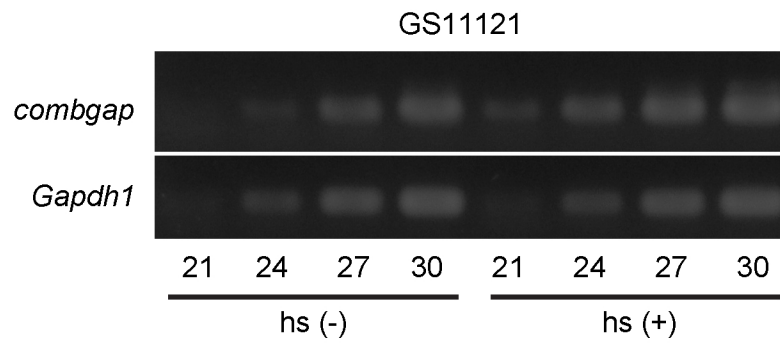

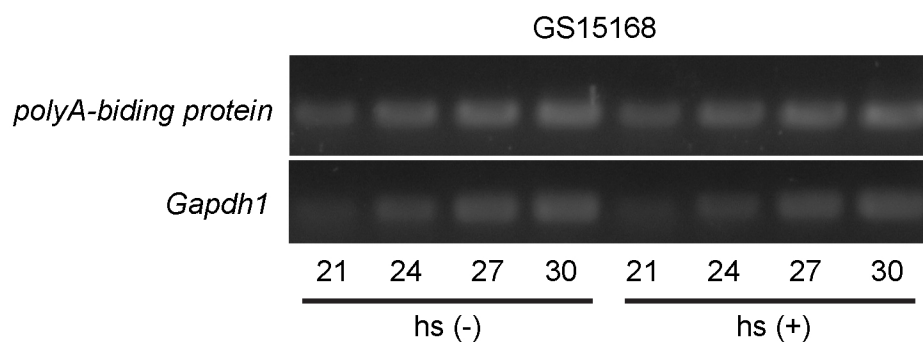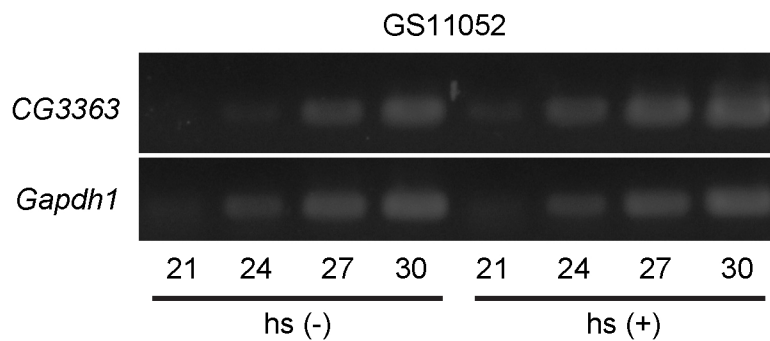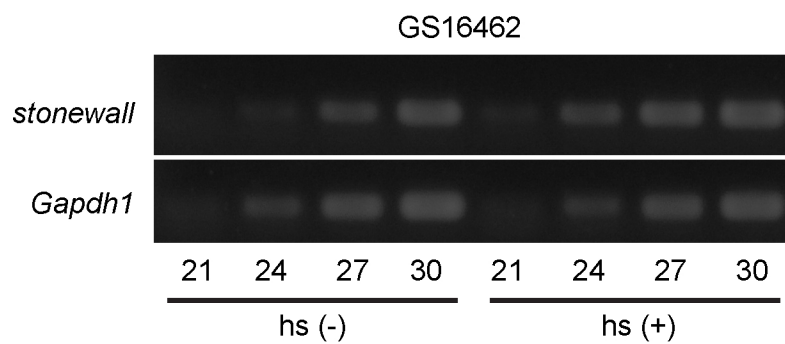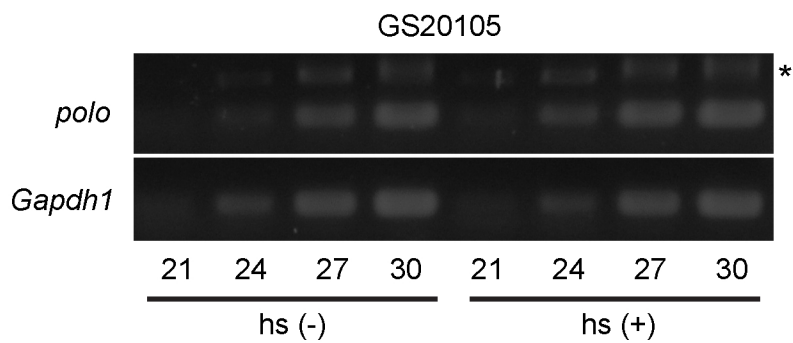

Supplement: Additional file 2: Figure S1 — Semi-quantitative RT-PCR confirmed the misexpression of reduced-lifespan genes upon heat-shock treatment. Results of semi-quantitative RT-PCR detecting cDNA fragments of Transportin, CG10277, Calpain-A, combgap, polyA-binding protein, CG3363, stonewall, and polo are shown. Total RNA was isolated from adult flies carrying hs-GAL4 and a GS insertion that were subjected to the same culture conditions used in our secondary screen, with or without heat-shock treatment. The numbers 21, 24, 27, and 30 are PCR cycles. Heat shock markedly increased the RT-PCR products from Transportin, CG10277, combgap, CG3363, polo, and stonewall. Glyceraldehyde 3 phosphate dehydrogenase 1 (Gapdh1) was used as an internal control. polo gave an additional RT-PCR product (shown by an asterisk). [file 1471-2156-15-46-S2.pdf]

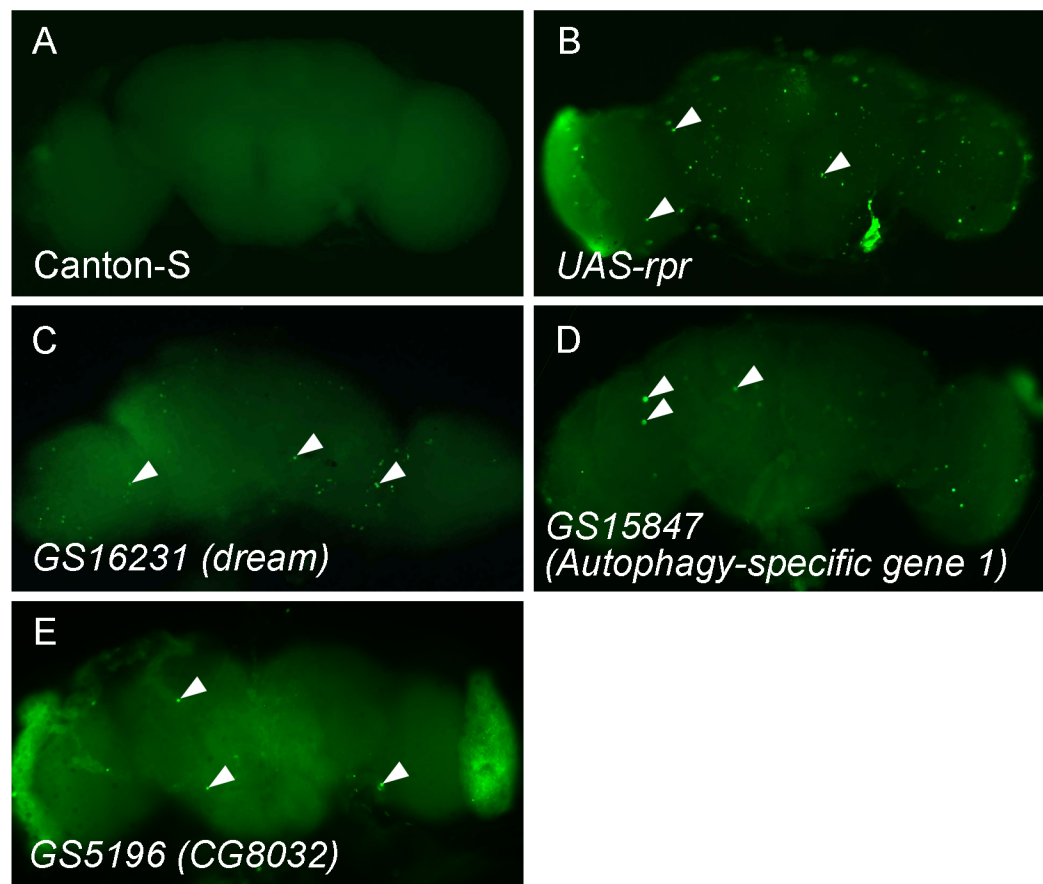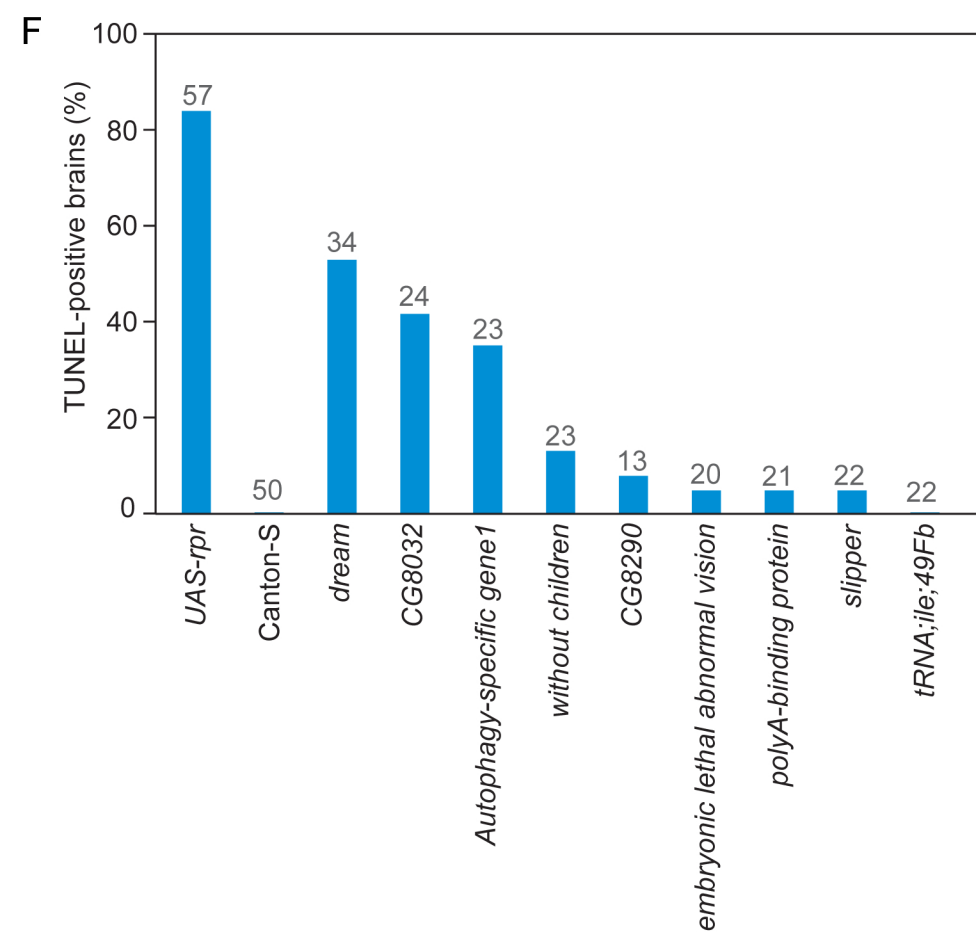

Supplement: Additional file 4: Figure S3 — The adult-specific misexpression of reduced-lifespan genes induced apoptosis in the adult brain. Canton-S (wild-type), UAS-rpr, and positive GS lines were crossed with hs-GAL4, and the F1 flies were heat-shocked as in the primary and secondary screens. Brains were dissected from flies that were still alive after more than half of the heat-shocked flies had died, and apoptotic cells were detected by TUNEL assay. Fluorescence microscopy images of adult brains are shown. Canton-S (A) and UAS-rpr(B) were used as negative and positive controls, respectively. (C-E) Representative samples of positive GS lines, GS16231 (dream) (C), GS15847 (Autophagy-specific gene 1) (D), and GS5196 (CG8032) (E), are shown. White arrowheads indicate TUNEL-positive apoptotic cells. (F) Frequency of TUNEL-positive brains is shown as a percentage. The number of samples tested is shown above each bar. [file 1471-2156-15-46-S4.pdf]
