# Supplementary material for: A gain-of-function screen to identify genes that reduce lifespan in the adult of Drosophila melanogaster
Source: BMC Genet. 2014 Apr 16;15:46. doi: 10.1186/1471-2156-15-46 (PMC4021436; doi:10.1186/1471-2156-15-46)
Supplement: Additional file 3: Figure S2 — Survival curves of adult flies expressing UAS-p35 (magenta), which encodes an apoptosis inhibitor, or UAS-GFP (green), in combination with each reduced-lifespan gene (shown at the top of each graph). Details are described in Figure 3. [file 1471-2156-15-46-S3.pdf]

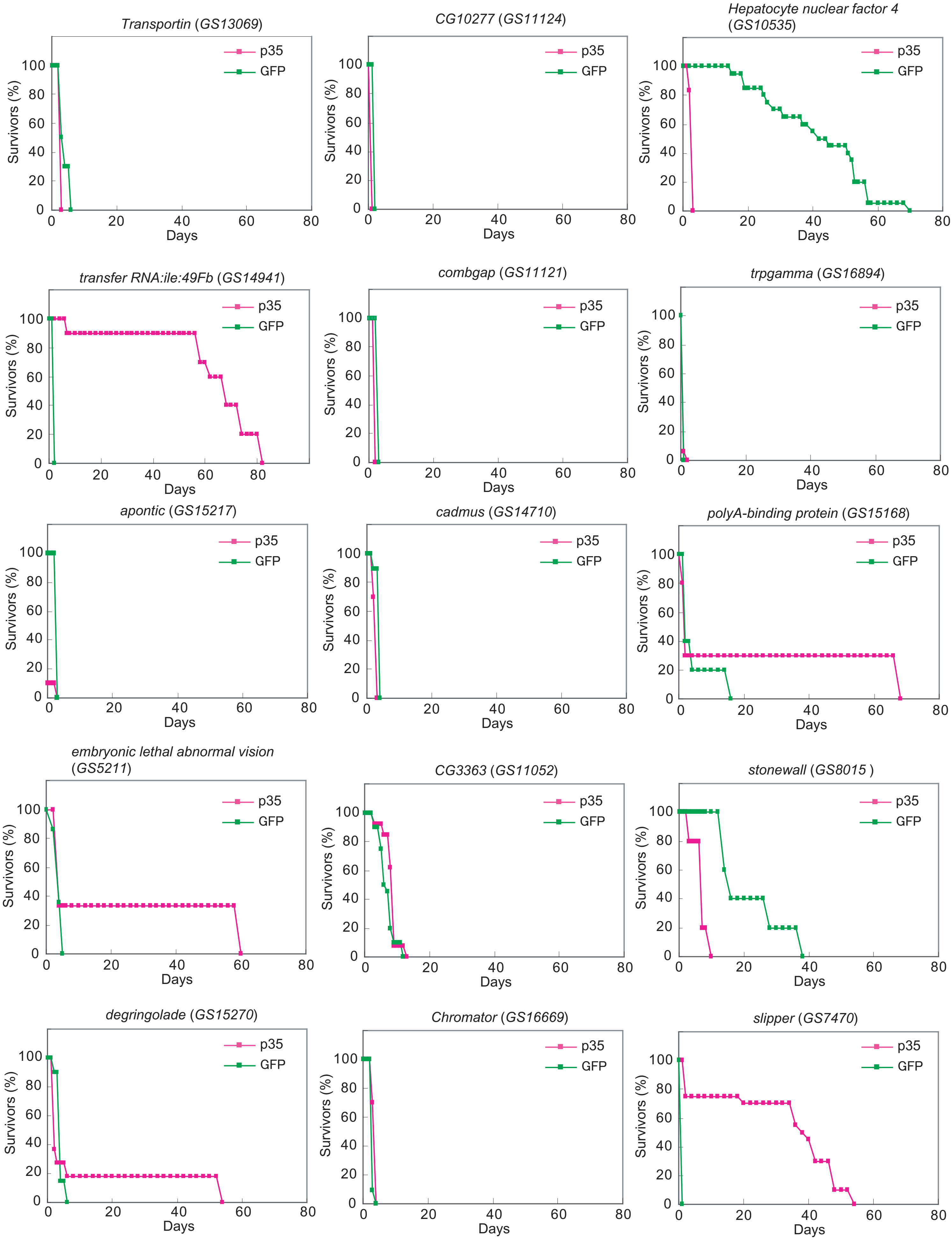
Transportin (GS13069)
CG10277 (GS11124)
Hepatocyte nuclear factor 4 (GS10535)
transfer RNA:ile:49Fb (GS14941)
combgap (GS11121)
trpgamma (GS16894)
apontic (GS15217)
cadmus (GS14710)
polyA-binding protein (GS15168)
embryonic lethal abnormal vision (GS5211)
CG3363 (GS11052)
stonewall (GS8015)
degringolade (GS15270)
Chromator (GS16669)
slipper (GS7470)
p35
GFP
Survivors (%)
Days
0
20
40
60
80
100

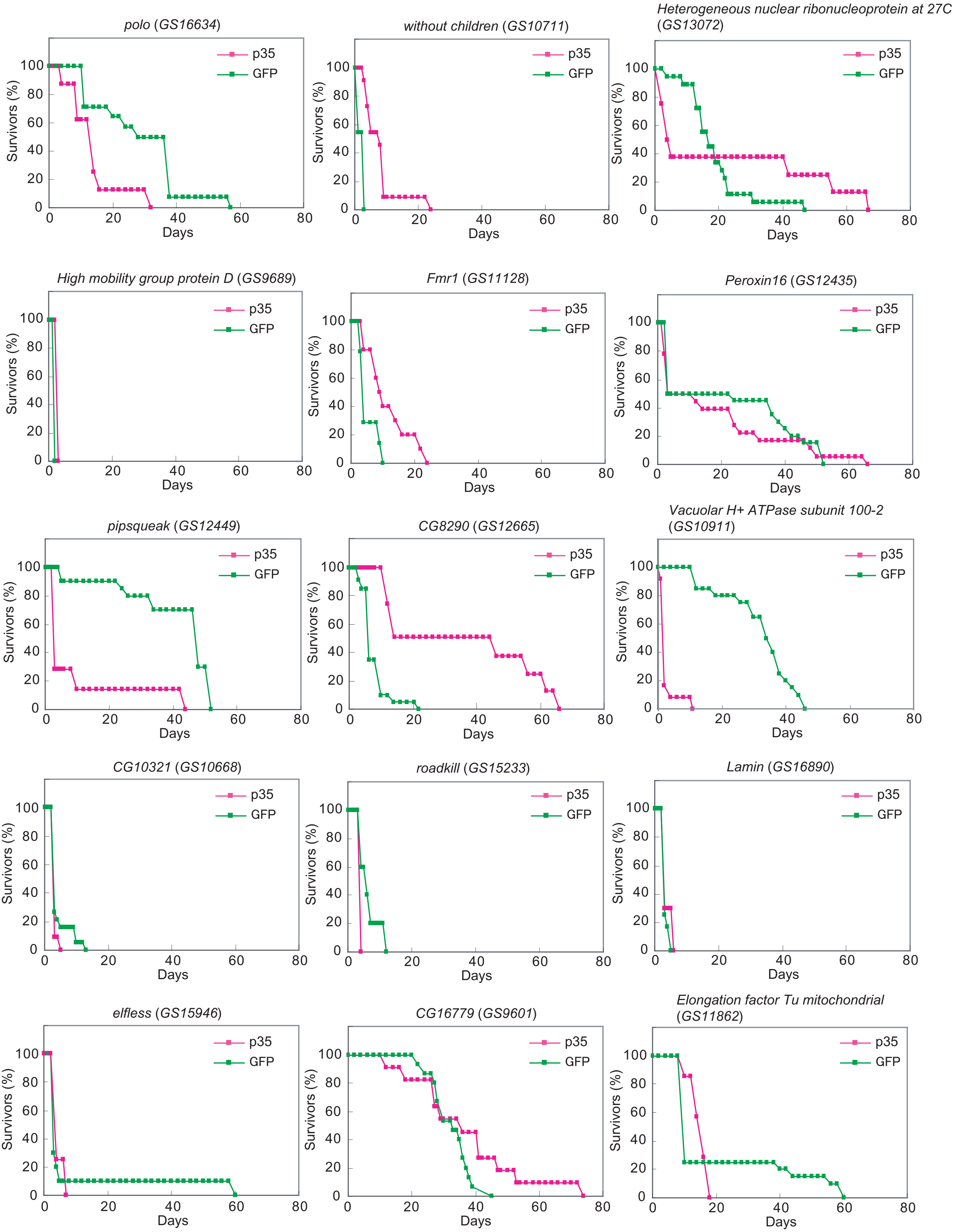

polo (*GS16634*)
without children (*GS10711*)
Heterogeneous nuclear ribonucleoprotein at 27C (*GS13072*)
High mobility group protein D (*GS9689*)
Fmr1 (*GS11128*)
Peroxin16 (*GS12435*)
pipsqueak (*GS12449*)
CG8290 (*GS12665*)
Vacuolar H+ ATPase subunit 100-2 (*GS10911*)
CG10321 (*GS10668*)
roadkill (*GS15233*)
Lamin (*GS16890*)
elfless (*GS15946*)
CG16779 (*GS9601*)
Elongation factor Tu mitochondrial (*GS11862*)
p35
GFP
Survivors (%)
Days
0
20
40
60
80
100

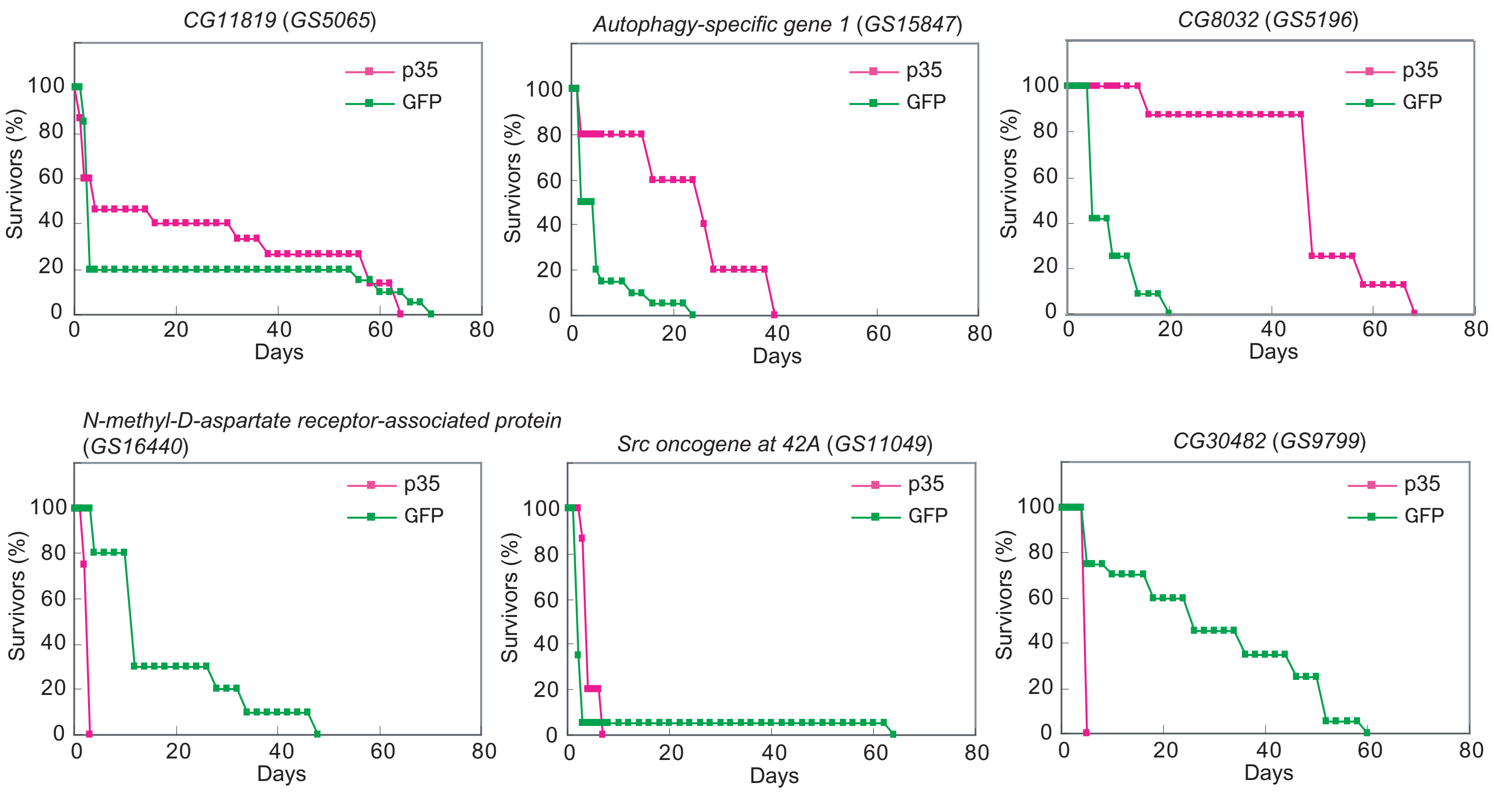
CG11819 (GS5065)
Autophagy-specific gene 1 (GS15847)
CG8032 (GS5196)
N-methyl-D-aspartate receptor-associated protein (GS16440)
Src oncogene at 42A (GS11049)
CG30482 (GS9799)
p35
GFP
Survivors (%)
Days
0
20
40
60
80
100
