# Supplementary material for: A gain-of-function screen to identify genes that reduce lifespan in the adult of Drosophila melanogaster
Source: BMC Genet. 2014 Apr 16;15:46. doi: 10.1186/1471-2156-15-46 (PMC4021436; doi:10.1186/1471-2156-15-46)
Supplement: Additional file 5: Figure S4 — Tissue-specific expression of GAL4 in the adult brain, gut, and Malpighian tubule in various GAL4 lines. We analyzed the GAL4 expression patterns in six lines (indicated at left). The UAS-GFP expression driven by each GAL4 line was analyzed by confocal microscopy after immunostaining for the GFP protein (green) in the adult brain and in the gut /Malpighian tubule. Corresponding optical microscopy images are shown at right. Arrows indicate visceral muscles; arrowheads show the Malpighian tubule. The regions outlined by white broken lines are magnified in panels to the immediate right. The relative intensity of GFP expression in various GAL4 lines is indicated in the bottom table, as - (negative), +/− (almost negligible), + (weak), ++ (medium), and +++ (strong). [file 1471-2156-15-46-S5.pdf]

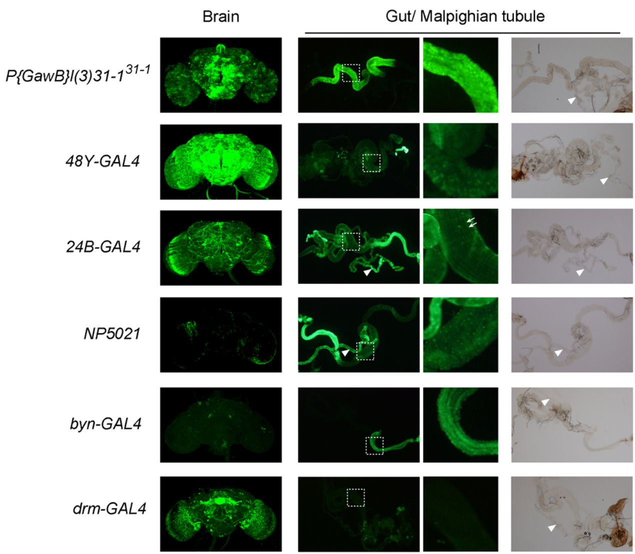

	Brain	Gut epithelium	Visceral muscle	Malpighian tubule
P{GawB}l(3)31-1$^{31\text{-}1}$	++	+++	-	-
48Y-GAL4	+++	+	-	-
24B-GAL4	+	-	++	+++
NP5021	+/-	++	-	+++
byn-GAL4	+/-	+ (hindgut)	-	+/-
drm-GAL4	+	+/-	-	-
