# Supplementary material for: A gain-of-function screen to identify genes that reduce lifespan in the adult of Drosophila melanogaster
Source: BMC Genet. 2014 Apr 16;15:46. doi: 10.1186/1471-2156-15-46 (PMC4021436; doi:10.1186/1471-2156-15-46)
Supplement: Additional file 6: Figure S5 — Tissue-specific expression experiments. Survival curves of adult flies misexpressing each reduced-lifespan gene. Tissue-specific expression was induced in adult flies with the TARGET system, and survivor rates were scored every three days. Details are described in Figure 4. [file 1471-2156-15-46-S6.pdf]

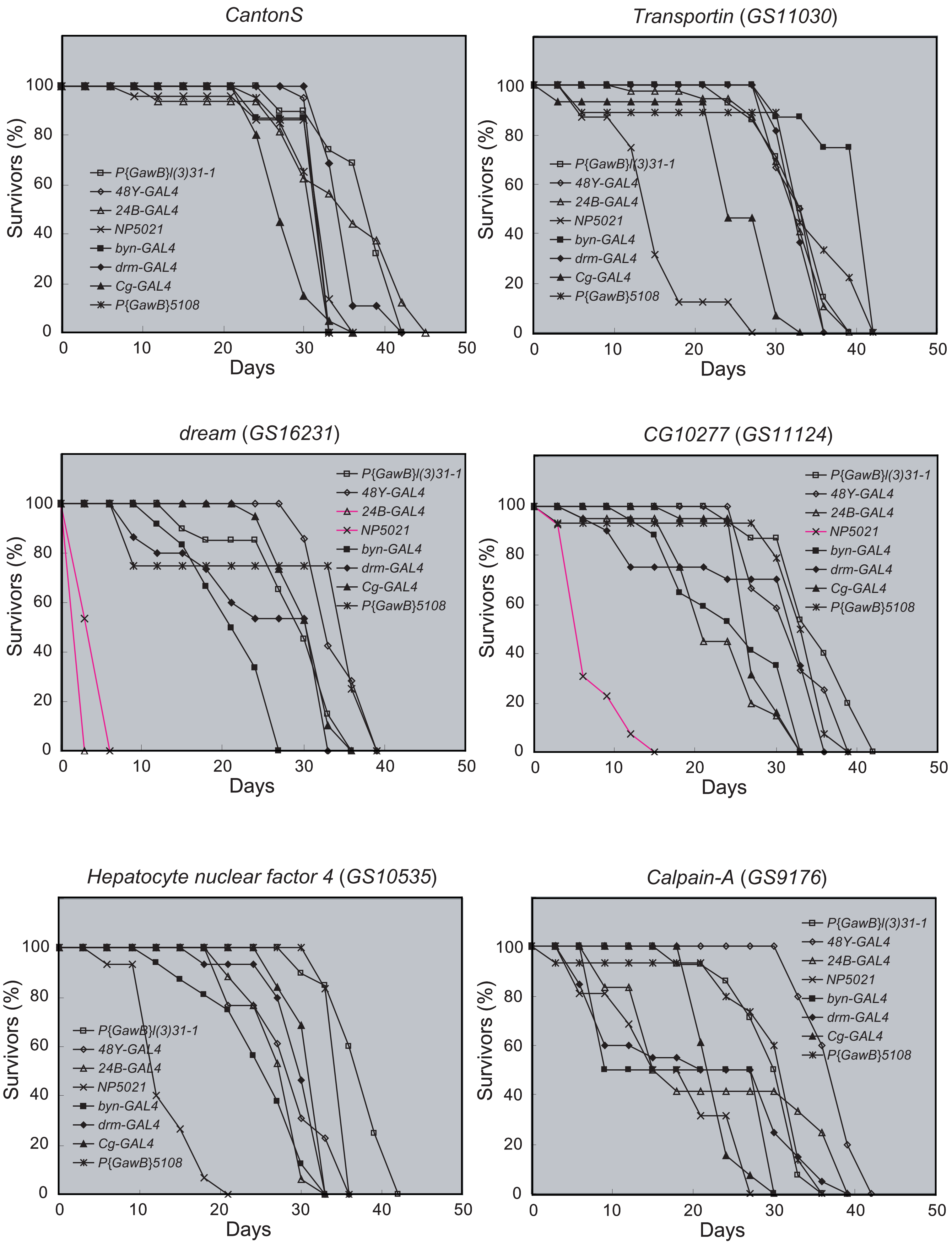
CantonS
Transportin (GS11030)
dream (GS16231)
CG10277 (GS11124)
Hepatocyte nuclear factor 4 (GS10535)
Calpain-A (GS9176)
Survivors (%)
Days
P{GawB}l(3)31-1
48Y-GAL4
24B-GAL4
NP5021
byn-GAL4
drm-GAL4
Cg-GAL4
P{GawB}5108
0
20
40
60
80
100
0
10
20
30
40
50

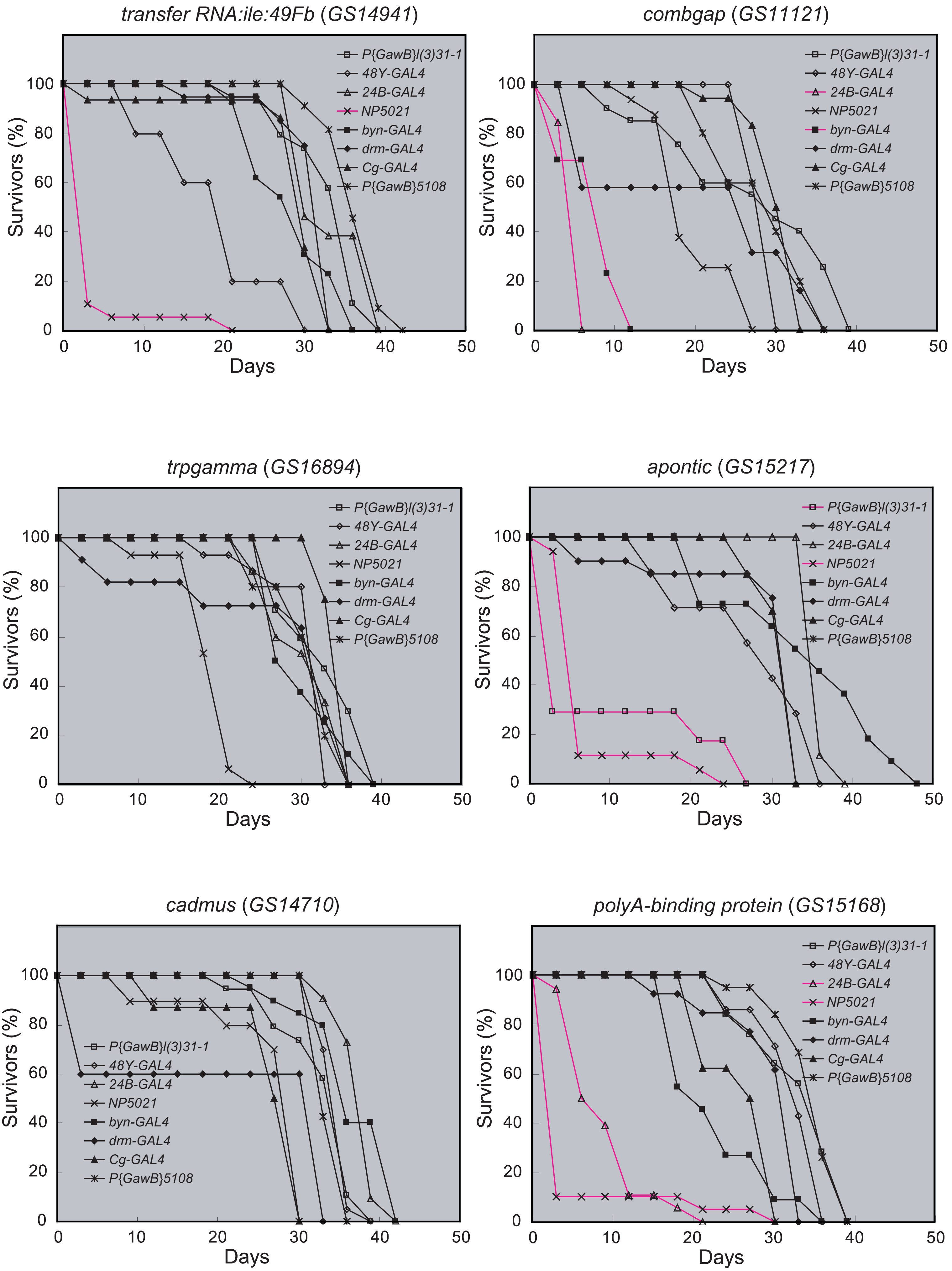
transfer RNA:ile:49Fb (GS14941)
combgap (GS11121)
trpgamma (GS16894)
apontic (GS15217)
cadmus (GS14710)
polyA-binding protein (GS15168)
Survivors (%)
Days
0
10
20
30
40
50
0
20
40
60
80
100
P{GawB}l(3)31-1
48Y-GAL4
24B-GAL4
NP5021
byn-GAL4
drm-GAL4
Cg-GAL4
P{GawB}5108

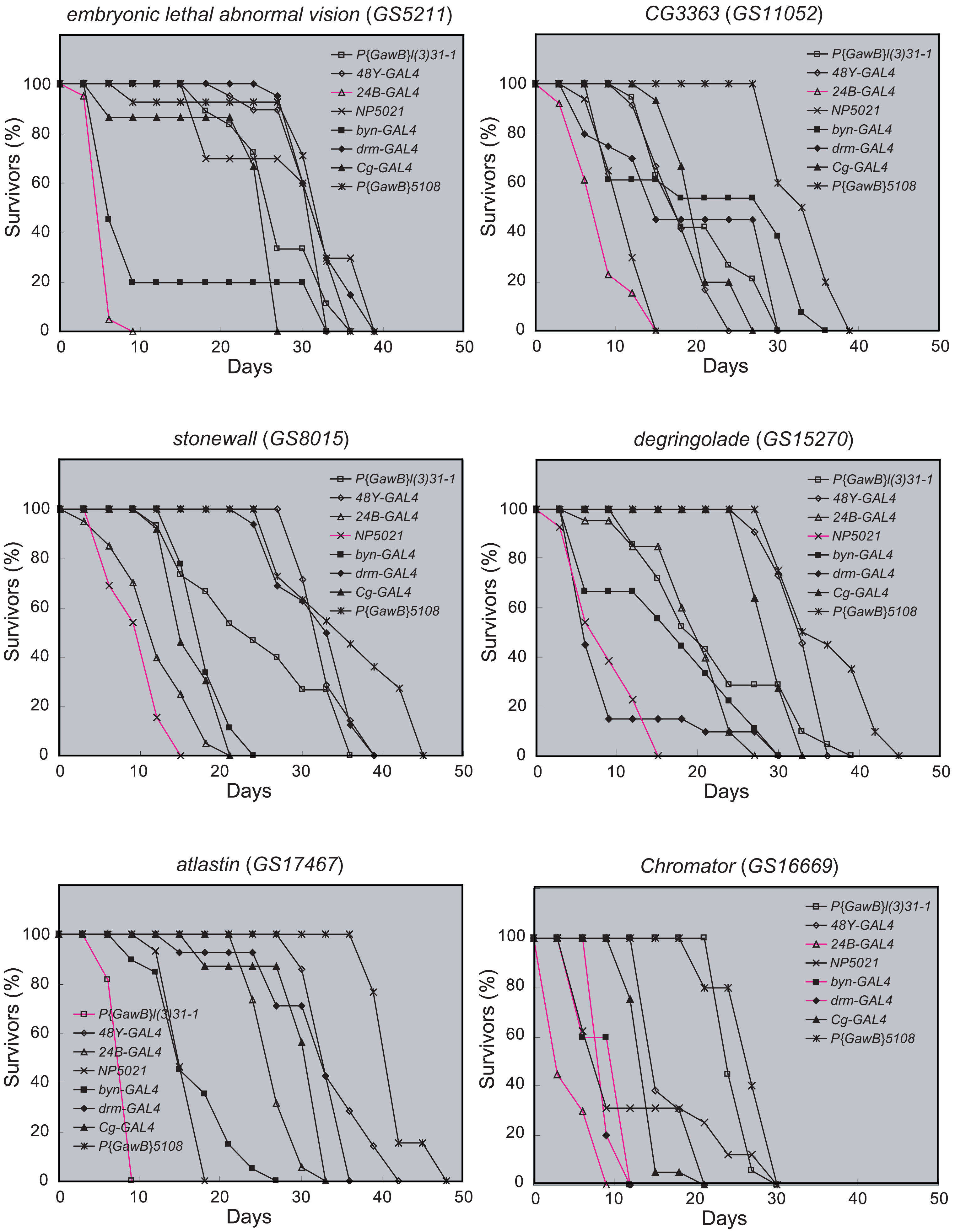
embryonic lethal abnormal vision (GS5211)
CG3363 (GS11052)
stonewall (GS8015)
degringolade (GS15270)
atlastin (GS17467)
Chromator (GS16669)
P{GawB}l(3)31-1
48Y-GAL4
24B-GAL4
NP5021
byn-GAL4
drm-GAL4
Cg-GAL4
P{GawB}5108
Survivors (%)
Days
0
20
40
60
80
100
0
10
20
30
40
50

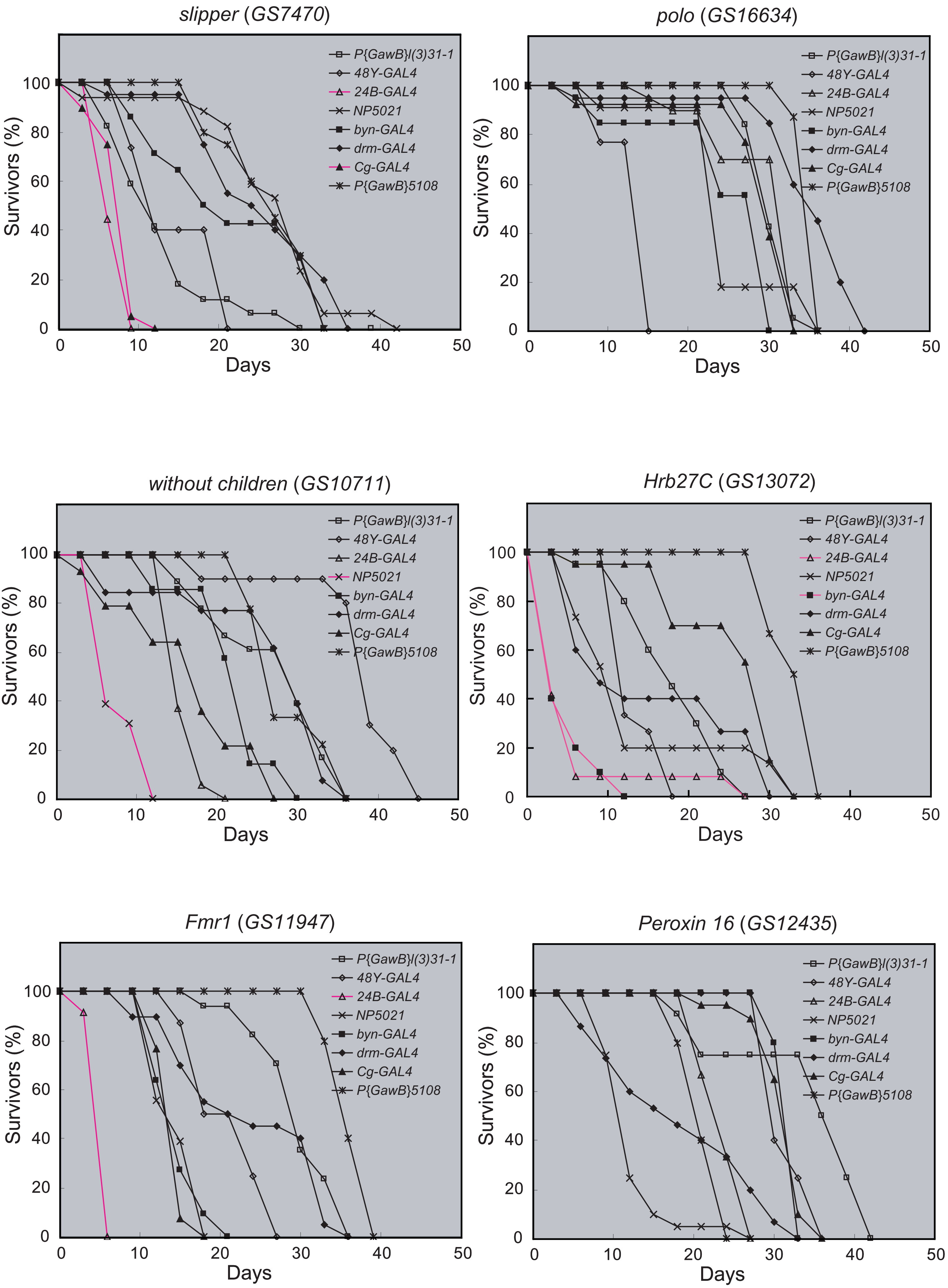

slipper (GS7470)
polo (GS16634)
without children (GS10711)
Hrb27C (GS13072)
Fmr1 (GS11947)
Peroxin 16 (GS12435)
P{GawB}l(3)31-1
48Y-GAL4
24B-GAL4
NP5021
byn-GAL4
drm-GAL4
Cg-GAL4
P{GawB}5108
Survivors (%)
Days
0
20
40
60
80
100
0
10
20
30
40
50

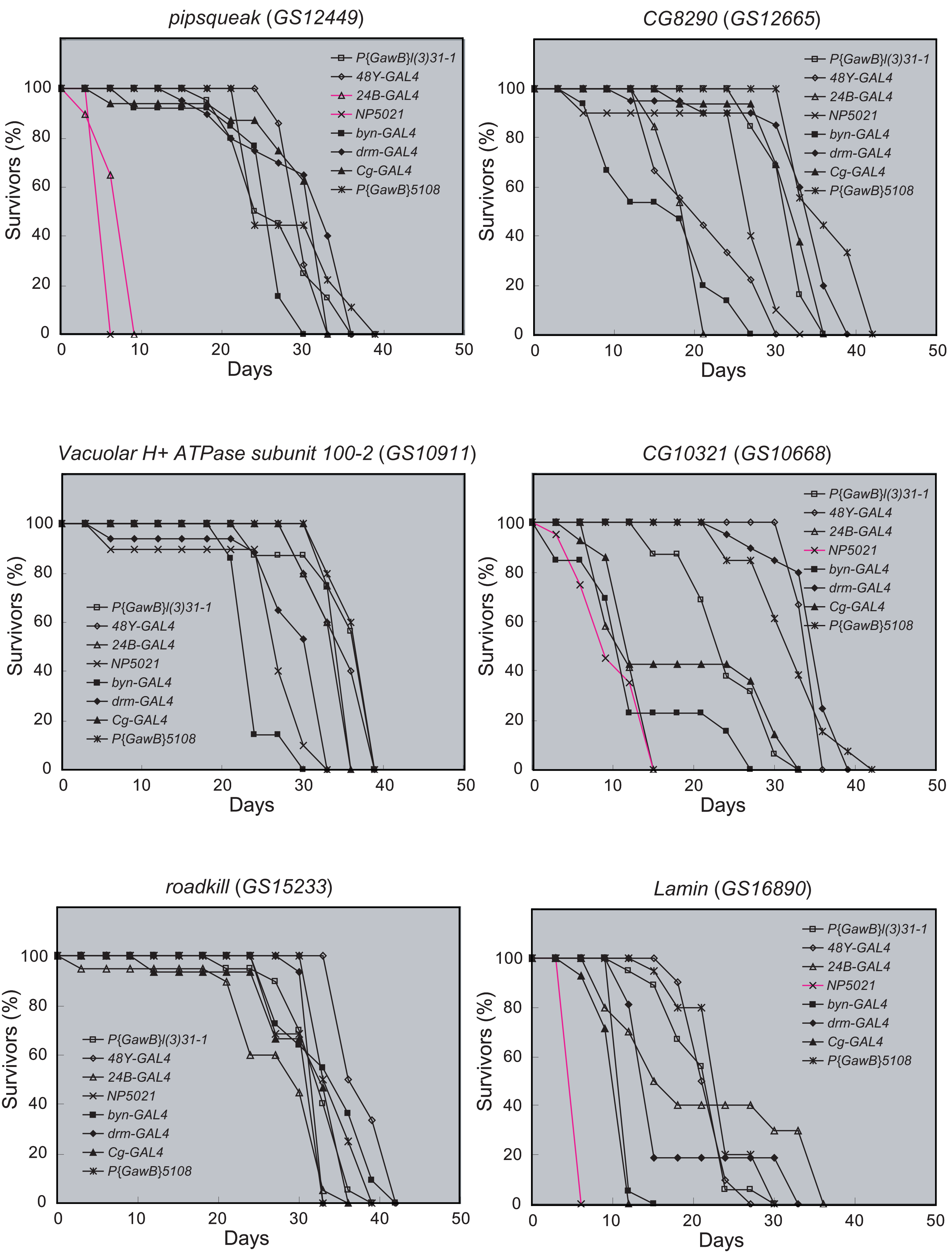

pipsqueak (GS12449)
CG8290 (GS12665)
Vacuolar H+ ATPase subunit 100-2 (GS10911)
CG10321 (GS10668)
roadkill (GS15233)
Lamin (GS16890)
Survivors (%)
Days
0
20
40
60
80
100
0
10
20
30
40
50
P{GawB}l(3)31-1
48Y-GAL4
24B-GAL4
NP5021
byn-GAL4
drm-GAL4
Cg-GAL4
P{GawB}5108

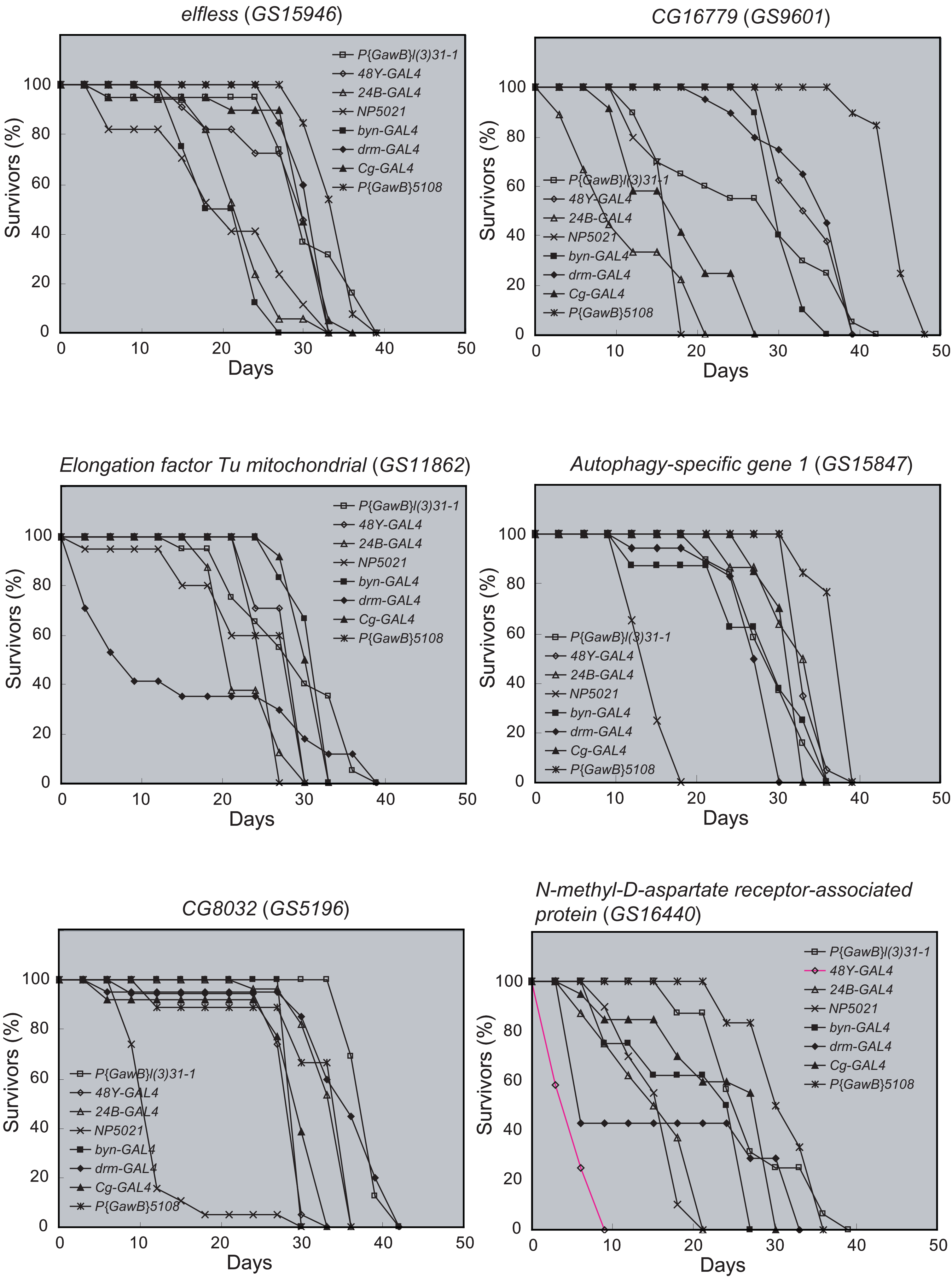
elfless (GS15946)
Survivors (%)
Days
P{GawB}l(3)31-1
48Y-GAL4
24B-GAL4
NP5021
byn-GAL4
drm-GAL4
Cg-GAL4
P{GawB}5108
CG16779 (GS9601)
Survivors (%)
Days
P{GawB}l(3)31-1
48Y-GAL4
24B-GAL4
NP5021
byn-GAL4
drm-GAL4
Cg-GAL4
P{GawB}5108
Elongation factor Tu mitochondrial (GS11862)
Survivors (%)
Days
P{GawB}l(3)31-1
48Y-GAL4
24B-GAL4
NP5021
byn-GAL4
drm-GAL4
Cg-GAL4
P{GawB}5108
Autophagy-specific gene 1 (GS15847)
Survivors (%)
Days
P{GawB}l(3)31-1
48Y-GAL4
24B-GAL4
NP5021
byn-GAL4
drm-GAL4
Cg-GAL4
P{GawB}5108
CG8032 (GS5196)
Survivors (%)
Days
P{GawB}l(3)31-1
48Y-GAL4
24B-GAL4
NP5021
byn-GAL4
drm-GAL4
Cg-GAL4
P{GawB}5108
N-methyl-D-aspartate receptor-associated protein (GS16440)
Survivors (%)
Days
P{GawB}l(3)31-1
48Y-GAL4
24B-GAL4
NP5021
byn-GAL4
drm-GAL4
Cg-GAL4
P{GawB}5108

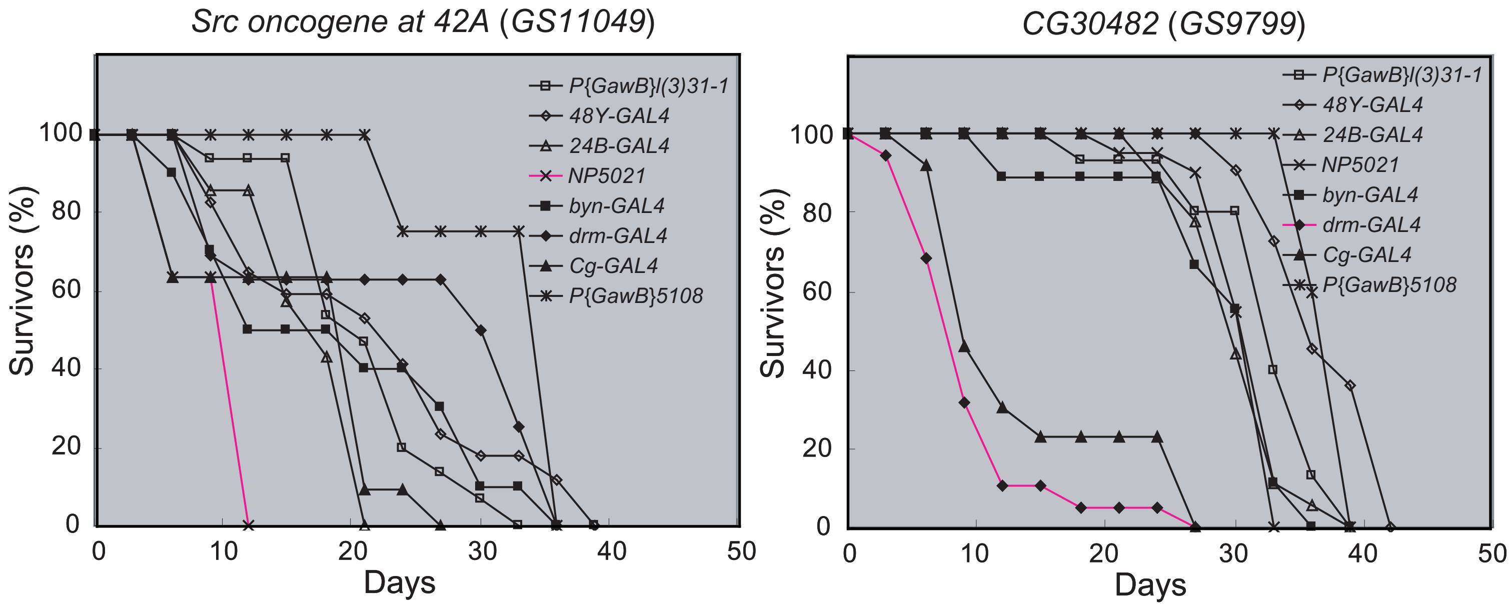
Src oncogene at 42A (GS11049)
P{GawB}l(3)31-1
48Y-GAL4
24B-GAL4
NP5021
byn-GAL4
drm-GAL4
Cg-GAL4
P{GawB}5108
Survivors (%)
Days
CG30482 (GS9799)
P{GawB}l(3)31-1
48Y-GAL4
24B-GAL4
NP5021
byn-GAL4
drm-GAL4
Cg-GAL4
P{GawB}5108
Survivors (%)
Days
